# A novel and evolutionarily distinct flavoprotein monooxygenase drives skatole degradation in *Rhodococcus*

**DOI:** 10.1128/aem.00228-26

**Published:** 2026-03-25

**Authors:** Nan Meng, Genxiu Li, Jiaxin Zhang, Jingwen Zhu, Xutong Kang, Jiahao Duan, Qiao Ma

**Affiliations:** 1 Institute of Environmental Systems Biology, College of Environmental Science and Engineering, Dalian Maritime University 12421, Dalian, China; Shanghai Jiao Tong University, Shanghai, China

**Keywords:** skatole, *Rhodococcus*, biodegradation, functional gene, flavoprotein monooxygenase

## Abstract

Skatole (3-methylindole), a persistent and toxic *N*-heterocyclic aromatic pollutant, poses significant bioremediation challenges due to poorly defined biodegradation processes. This study systematically investigated the genetic determinants and metabolic mechanisms of skatole degradation in the environmentally versatile Gram-positive genus *Rhodococcus*. We demonstrated that skatole degradation was a broadly conserved trait across diverse *Rhodococcus* species, including *R. aetherivorans*, *R. pyridinivorans*, *R. ruber*, and *R. qingshengii*. Genomic and functional analyses uncovered a novel flavoprotein monooxygenase (FPMO) SkaA as the initial catalyst for skatole catabolism. Heterologous expression in *Escherichia coli* confirmed SkaA’s essential catalytic role, with high-resolution liquid chromatography-tandem mass spectrometry identifying the key products 3-methyloxindole and 3-hydroxy-3-methyloxindole. Distribution studies revealed SkaA homologs predominantly in Actinobacteria, particularly *Nocardia* and *Rhodococcus*, indicating metabolic capacity for skatole transformation within this phylum. Notably, *Rhodococcus* strains lacking the *skaA* gene also retained skatole degradation activity, implying the existence of alternative genetic determinants. Crucially, phylogenetic analysis positioned SkaA as a distinct subclass within Group E FPMOs, exhibiting ≤40% sequence identity to reported styrene/indole oxygenases. The phylogenetic segregation of skatole, indole, and styrene monooxygenases provides a predictive framework for functional annotation of Group E FPMOs. Our findings elucidate a novel skatole transformation pathway, establish *Rhodococcus* as environmentally versatile biocatalysts, and provide new insights into the study of Group E FPMOs.

**IMPORTANCE:**

Skatole, a notorious malodorous compound, is mainly produced via the microbial anaerobic degradation of tryptophan. Its presence raises significant environmental and public health concerns. Yet, the enzymes responsible for its biodegradation remain poorly characterized. This study demonstrates that SkaA catalyzes the initial oxidation step in skatole metabolism. Distribution analysis reveals that SkaA homologs are predominantly in Actinobacteria, especially within the genera *Rhodococcus* and *Nocardia*. Phylogenetic analysis positions SkaA in a novel subclass within Group E flavoprotein monooxygenases (FPMOs). These findings not only provide crucial insights into skatole metabolism but also expand our understanding of the functional diversity of Group E FPMOs.

## INTRODUCTION

Odorous emissions, comprising complex mixtures of inorganic (e.g., H_2_S and NH_3_) and organic pollutants (e.g., volatile fatty acids, cresols, and indoles), pose significant challenges to environmental quality, animal welfare, and public health across livestock, aquaculture, landfill, and wastewater treatment sectors (1–4). Among these compounds, skatole (3-methylindole) is distinguished by its exceptional recalcitrance and malodor, attributable to its exceptionally low odor threshold (0.327 ng/L, as determined by expert panels), environmental persistence, and documented cytotoxicity (5). This tryptophan-derived microbial metabolite can accumulate to concerning levels (e.g., up to 20 mg/L in wastewater) driven by anaerobic bacteria, such as *Clostridium* and *Olsenella* (6–8). Although recent advances have elucidated its biosynthetic pathways, the environmental fate and transformation mechanisms of skatole remain poorly understood, hindering the development of effective mitigation strategies (9).

Microbial degradation represents a promising approach for skatole remediation, with diverse taxa reported to metabolize this compound. Current studies have focused predominantly on aerobic degradation, identifying several Gram-negative genera (e.g., *Pseudomonas*, *Acinetobacter*, *Cupriavidus*, *Rhodopseudomonas*, and *Burkholderia*) capable of skatole catabolism, with tentative pathways proposed (10–18). Conversely, gram-positive degraders are less characterized, with reports largely restricted to *Rhodococcus* strains. *Rhodococcus* species are robust, versatile, and metabolically redundant degraders of aromatic pollutants (19–24). Their capacity to colonize diverse niches (e.g., soil and sludge) further positions this genus as an ideal candidate for bioremediation applications. Our previous metagenomic and metatranscriptomic analyses identified *Rhodococcus* as a primary population actively responsible for skatole degradation in activated sludge systems (20). We further demonstrated the skatole degradation capacity and efficacy in a newly isolated strain *R. aetherivorans* DMU1 (22). Subsequent studies have since confirmed degradation capabilities in other strains, including *R. aetherivorans* BCP1 and *R. ruber* R1 (24). These collective advances prompt investigation into whether skatole biodegradation constitutes a conserved catabolic trait within the *Rhodococcus* genus.

Despite progress in skatole-degrading strain isolation and characterization, critical knowledge gaps persist. First, the genetic basis for skatole degradation remains unconfirmed. Our pioneering work on *R. aetherivorans* DMU1 identified a potential oxidoreductase gene cluster involved in skatole conversion via RNA-seq and quantitative PCR analyses (22). This cluster contained a gene encoding a putative flavoprotein monooxygenase (FPMO) putatively involved in skatole biodegradation. Second, the biotransformation pathway of skatole remains unclear. Although several intermediates have been tentatively identified via mass spectrometry, their structural assignments remain ambiguous, and the enzymatic steps involved are undefined (20, 22).

In this study, we systematically investigated skatole degradation using environmentally versatile *Rhodococcus* strains. The objectives were to address the following questions. (i) Is skatole degradation a common trait among *Rhodococcus* strains? (ii) What is the metabolic pathway for skatole degradation? (iii) What is the core functional gene responsible, and how is it distributed? Our findings should provide new insights into the molecular mechanisms of skatole metabolism in gram-positive bacteria and identify novel biocatalytic resources for environmental remediation.

## RESULTS

### Skatole degradation is prevalent in *Rhodococcus* strains

All the reported skatole-degrading bacteria were summarized in Table S1. Skatole degradation has been predominantly investigated in different gram-negative bacteria, including *Pseudomonas*, *Acinetobacter*, *Burkholderia*, and *Cupriavidus*. Notably, *Rhodococcus*, a genus of typical gram-positive bacteria, possesses large genomes with redundant aromatic-degrading genes. Such genetic architecture underpins their metabolic versatility and makes them attractive microbial resources for bioremediation (25). Notably, recent studies have identified several *Rhodococcus* species as highly efficient skatole degraders, including *R. aetherivorans* DMU1, *R. pyridinivorans* Rp3, and *R. ruber* R1 (21–24). These findings motivated us to assess whether skatole degradation capability is a prevalent trait within this genus.

The skatole-degrading capacity of eight *Rhodococcus* strains, previously isolated in our laboratory from activated sludge and marine sediment samples, was assessed (19). Degradation performance was evaluated using two culture media: MS (skatole as the sole carbon source) and MSY (supplemented with yeast extract as an additional carbon source) (Table 1). In MS medium, strains DMU1, DMU2, and SJ-2 demonstrated the highest activity, achieving complete skatole removal within 48 h, and strains DMU114 and DMU2021 required 96 h to reach >99% degradation (Fig. S1). Conversely, strains E7 (27.7%, 96 h), SJ-1 (34.0%), and SJ-3 (6.1%) exhibited weaker degradation activity. In MSY medium, five strains (DMU1, DMU114, SJ-1, SJ-2, and SJ-3) degraded >99% of skatole within 96 h (Fig. S2). Notably, strains SJ-1 and SJ-3, which performed poorly in MS, achieved complete removal within 48 h in MSY. Strain DMU114 also degraded skatole faster in MSY (77.5% at 48 h) than in MS (11.0% at 48 h), suggesting that yeast extract enabled co-metabolism. Conversely, the degradation efficiency of strains DMU2, DMU2021, and SJ-2 was lower in MSY than in MS, indicating potential catabolite repression. Overall, DMU1 demonstrated superior degradation capability under both conditions, while strain E7 consistently showed the least activity.

**TABLE 1:** Skatole-degrading performance of the *Rhodococcus* strains

Strain	MS-48 h	MS-96 h	MSY-48 h	MSY-96 h
DMU1	100%	100%	97.7%	100%
DMU2	100%	100%	71.2%	80.5%
DMU114	11.0%	100%	77.5%	100%
DMU2021	5.4%	99.2%	34.2%	53.7%
E7	19.5%	27.7%	24.2%	32.5%
SJ-1	12.2%	34.0%	100%	100%
SJ-2	100%	100%	76.8%	99.2%
SJ-3	3.7%	6.1%	100%	100%

Collectively, all tested *Rhodococcus* strains demonstrated the capacity to degrade skatole, albeit with performance being strain-specific and medium-dependent. This phenotypic variation led us to hypothesize the potential presence of conserved genetic determinants that enabled skatole degradation across the genus. To identify these mechanisms, we performed whole-genome sequencing of the strains followed by comparative genomic analysis.

### Genome feature of the *Rhodococcus* strains

Genome sequences for strains DMU1, DMU114, DMU2021, and SJ-1 were obtained from our previous studies (20, 26–28), and those for strains E7, DMU2, SJ-2, and SJ-3 were determined in this study. The genome features for all eight strains are summarized in Table 2. Genomes ranged in size from 5.2 to 6.8 Mb and possessed GC contents of 62.5–70.4%. A comparative genomic analysis was performed using a circular map, employing the genome of strain DMU1 as a reference. Genes were color-coded based on their similarity to orthologs in strain DMU1 (Fig. S3).

**TABLE 2:** Genome information of the *Rhodococcus* strains used in this study

Strain	Species	Source	GC content (%)	Genome size (bp)	Gene number	BioProject accession number
DMU1	*aetherivorans*	Activated sludge	70.0	6,835,256	6250	PRJNA616208
DMU2	*qingshengii*	Activated sludge	62.5	6,634,658	6498	PRJNA1295803
DMU114	*pyridinivorans*	Activated sludge	67.9	5,241,731	5066	PRJNA1267748
DMU2021	*pyridinivorans*	Activated sludge	67.7	5,511,496	5183	PRJNA753252
E7	*qingshengii*	Marine sediment	62.5	6,289,418	6042	PRJNA1295815
SJ-1	*ruber*	Marine sediment	70.4	5,738,084	5386	PRJNA1171201
SJ-2	Unknown	Marine sediment	65.4	5,773,915	5717	PRJNA1295737
SJ-3	Unknown	Marine sediment	65.4	5,833,229	5553	PRJNA1301114

Taxonomic classification of these strains was determined by whole-genome ANI and dDDH analyses (Tables S2 and S3). The ANI values between our strains and 45 representative *Rhodococcus* strains ranged from 69.6% to 98.8% (Fig. S4). Pairwise comparisons revealed that *R. aetherivorans* JCM 14343 exhibited the highest ANI (97.2%) and dDDH (91.4%) with strain DMU1, confirming its species affiliation. Strains DMU2 and E7 were most similar to *R. qingshengii* JCM 15477, with ANI values of 98.4% and 98.6%, and dDDH values of 88.5% and 69.5%, respectively. Strains DMU114 and DMU2021 shared the highest mutual ANI similarity (98.1%). DMU2021 showed the highest similarity to *R. pyridinivorans* DSM 44555 (ANI 98.4% and dDDH 91.1%), confirming its classification. Strain SJ-1 demonstrated the highest ANI (98.8%) and dDDH (92.7%) similarities with *R. ruber* DSM 43338, confirming classification of this species. Strains SJ-2 and SJ-3 were most similar to each other (>98%), whereas ANI values with all other investigated *Rhodococcus* species were below 95% and dDDH values were below 35%, and their species required further verification.

Based on these genomic analyses, the strains were assigned as follows: DMU1 as *R. aetherivorans*; DMU2 and E7 as *R. qingshengii*; DMU114 and DMU2021 as *R. pyridinivorans*; SJ-1 as *R. ruber*. Strains SJ-2 and SJ-3 were unknown species (Table 2). Three species, that is, *R. aetherivorans* (strain DMU1), *R. pyridinivorans* (strain Rp3), and *R. ruber* (strain R1), have been reported to catalyze skatole (19–22). Our study expanded the known degraders to include *R. qingshengii* and a potentially novel species, thereby identifying new microbial resources for skatole bioremediation. Notably, despite both being classified as *R. qingshengii*, strains E7 (<33% degradation in MS/MSY) and DMU2 (complete degradation within 48 h in MS medium) exhibited different skatole degradation efficiencies, further confirming the critical role of intra-species variation in degradation performance (Table 1).

### Functional verification of the skatole oxygenase gene in strain DMU1

A flavoprotein monooxygenase gene (*skaA*, 1,368 bp), previously identified in *R. aetherivorans* DMU1 via transcriptomic analysis, was implicated as the key enzyme involved in skatole degradation (22). In this study, qPCR analysis confirmed that gene *skaA* was significantly upregulated in response to both skatole (53.4-fold) and indole (162.5-fold), supporting its functional role in metabolizing both compounds (Fig. S5). The *skaA* gene was subsequently cloned and heterogeneously expressed in *Escherichia coli* BL21(DE3) strain (Fig. S6). Notably, the resulting recombinant strain was capable of skatole biotransformation, as well as indole conversion into indigoid pigments (Fig. S6).

### Skatole transformation product identification

**Fig 1:**
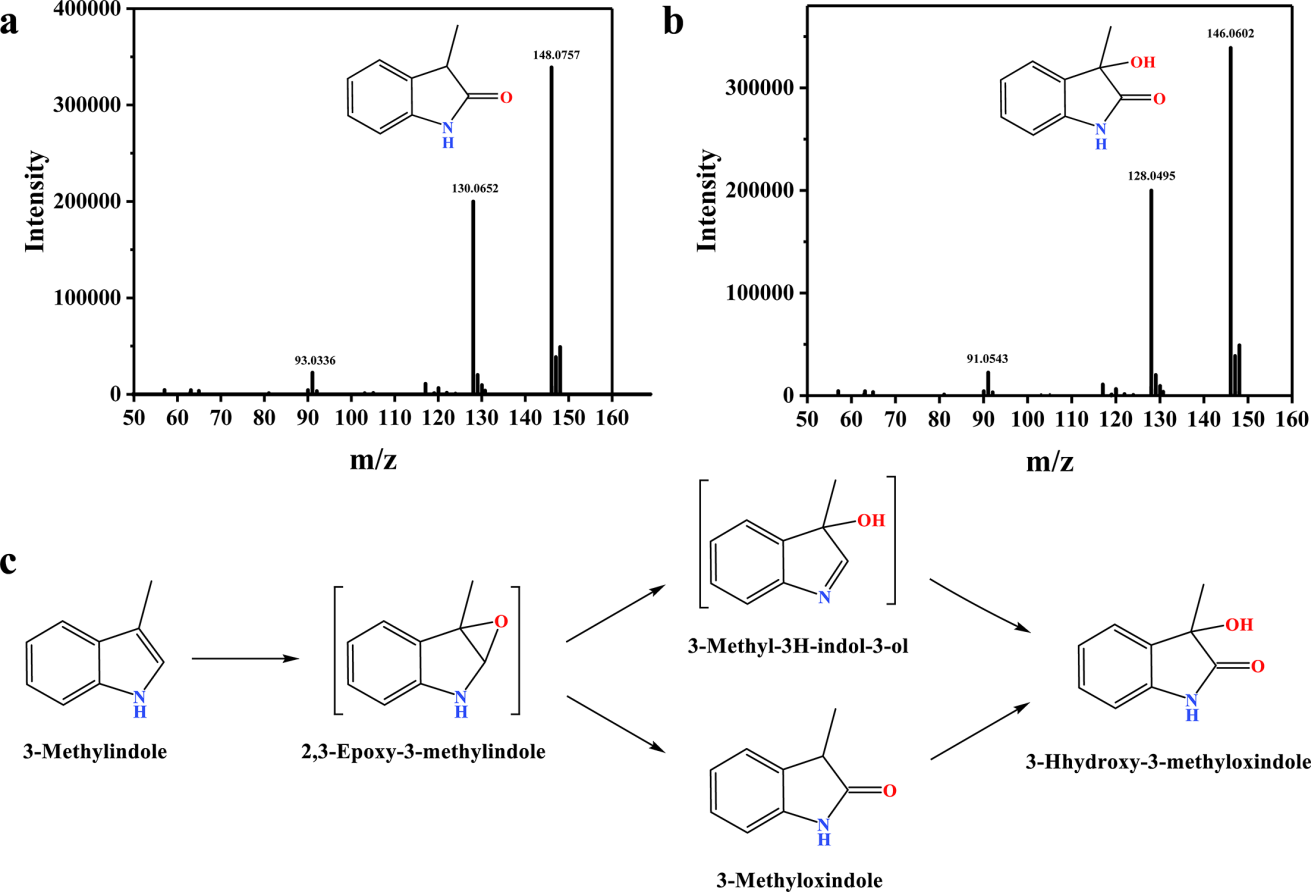
Identification of skatole transformation metabolites in the recombinant *E. coli* strain expressing DMU1-*skaA*. (**a**) Mass spectrum of product I (3-methyloxindole). (**b**) Mass spectrum of product II (3-hydroxy-3-methyloxindole). (**c**) Proposed pathway for skatole transformation.

Metabolite analysis was performed using resting cells, with the reaction mixtures analyzed by LC-HRMS. Three major products, each exhibiting an [M+H]^+^ ion at *m*/*z* 148.0757, were detected in positive ion mode with distinct retention times (RTs) of 5.37, 7.82, and 9.66 min. These compounds shared the molecular formula C_9_H_9_NO and yielded identical MS/MS fragmentation patterns, indicating they were structural isomers originating from the monooxygenation of skatole. Product I (RT 7.82 min) was identified as 3-methyloxindole by comparison with an authentic standard (Fig. 1a; Fig. S7a). The products eluting at 5.37 and 9.66 min were tentatively proposed as 3-methyl-3H-indol-3-ol and 2,3-epoxy-3-methylindole, respectively, with the latter being a characteristic epoxide intermediate formed by Group E FPMOs (29). Additionally, product II (RT 6.24 min, *m*/*z* 146.0602 [M+H]^+^) was confirmed as 3-hydroxy-3-methyloxindole by comparison with a reference standard (Fig. 1b; Fig. S7b). The purified SkaA enzyme exhibited relatively low activity. Nonetheless, metabolite analysis confirmed that its product profile was identical to that generated in resting cell experiments.

Collectively, these results suggested a novel skatole biotransformation pathway (Fig. 1c). We propose that skatole was initially oxygenated by SkaA to form the epoxide intermediate 2,3-epoxy-3-methylindole (Fig. S8). This unstable intermediate then underwent further transformations yielding 3-methyloxindole (RT 7.82 min), which was subsequently converted to 3-hydroxy-3-methyloxindole. Importantly, both 3-methyloxindole and 3-hydroxy-3-methyloxindole were also detected in metabolite analyses of the wild-type strain *R. aetherivorans* DMU1, confirming their relevance in the native metabolic pathway.

### Distribution and genomic architecture of skatole degradation genes

Genomic analysis revealed that the *skaA* gene was present in multiple *Rhodococcus* species used in this study (Table S4). Putative skatole monooxygenases (SkaMOs) exhibiting varying degrees of protein sequence identity (>30%) were identified in *R. aetherivorans* JCM 14343 (38.2% and 99.3%), *R. indonesiensis* SARSHI1 (38.4%), *R. opacus* ATCC 51881 (30.7%, 33.4%, and 37.9%), *R. sacchari* Z13 (36.9%), *R. spongiicola* LHW 50502 (31.9% and 34.0%), and *R. triatomae* DSM 44893 (36.6%). A *skaA* homolog gene was also detected in *R. ruber* SJ-1 (38.2%). However, no similar sequences were found in strains DMU2, DMU114, DMU2021, E7, SJ-2, and SJ-3. Strikingly, pairwise comparison showed the SkaA sequence from strain DMU1 shares low identity (<40%) with orthologs in all listed strains except its conspecific strain *R. aetherivorans* JCM 14343, indicating that this monooxygenase was primarily conserved within this species.

Analysis of all currently available skatole-degrader genomes (Table S5) revealed putative SkaMOs in *Rhodococcus* strains R1 (38.2% and 98.9%) and BCP1 (38.2% and 99.1%), as well as *Cupriavidus* sp. KK10 (32.8% and 34.8%), *Burkholderia* sp. IDO3 (32.2% and 35.7%), and *Acinetobacter piscicola* p38 (31.6% and 36.9%). Notably, the recently characterized degrader *R. pyridinivorans* Rp3 lacked detectable SkaMOs, a finding consistent with strains *R. pyridinivorans* DMU2021 and DMU114, suggesting the existence of novel degradation mechanisms within this species.

**Fig 2:**
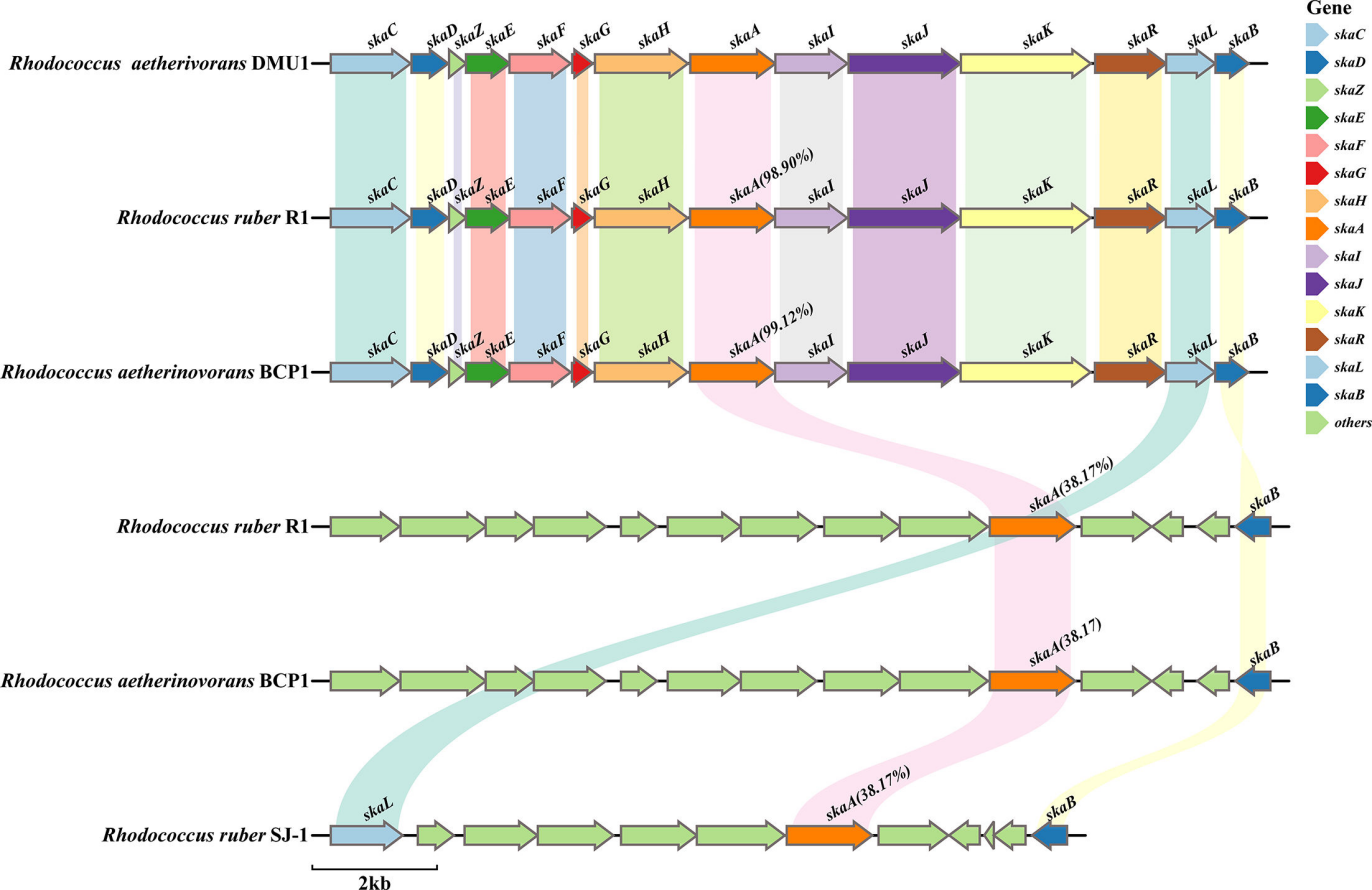
Gene cluster analysis of typical skatole-degrading *Rhodococcus* strains. Accession numbers for the strain DMU1 proteins are in Table S6, with the IDs of SkaA homologs from other strains provided in Table S5.

Comparative analysis of the *ska* gene clusters across *Rhodococcus* strains revealed a conserved, functionally modular architecture in key degraders, such as strains DMU1, R1, and BCP1 (20, 24). As illustrated in Fig. 2, this 14-gene cluster (*skaCDZEFGHAIJKRLB*) was transcriptionally upregulated under skatole stress in both strains DMU1 and R1 in previous studies (22, 24). Bioinformatic analysis indicated that the initial oxidation was likely mediated by the *skaAB*-encoded monooxygenase-reductase complex, which catalyzed skatole epoxidation. Here, SkaB, a predicted flavin reductase, was presumed to supply reducing equivalents to the oxygenase SkaA. Notably, a *skaB* gene (531 bp) was identified 7,058 bp downstream of *skaA*, rather than in an adjacent position in strain DMU1 (Fig. 2). Subsequent ring cleavage was likely executed by a three-component aromatic ring-hydroxylating oxygenase (ARHO) system (*skaCDFG*), while cluster expression was potentially governed by the embedded LuxR-type regulator *skaR* (Table S6).

While strain DMU1 possessed a single genomic copy of *skaA,* strains R1 and BCP1 harbored an additional SkaA homolog exhibiting low sequence similarity to SkaA_DMU1 (38.2%). This homolog was associated with a truncated gene cluster containing only *skaAB*, a configuration similar to that found in strain SJ-1 (Fig. 2). The specific roles of the two gene clusters in the degradation of skatole require further validation.

### Taxonomic distribution of SkaA in the RefSeq and NR databases

**Fig 3:**
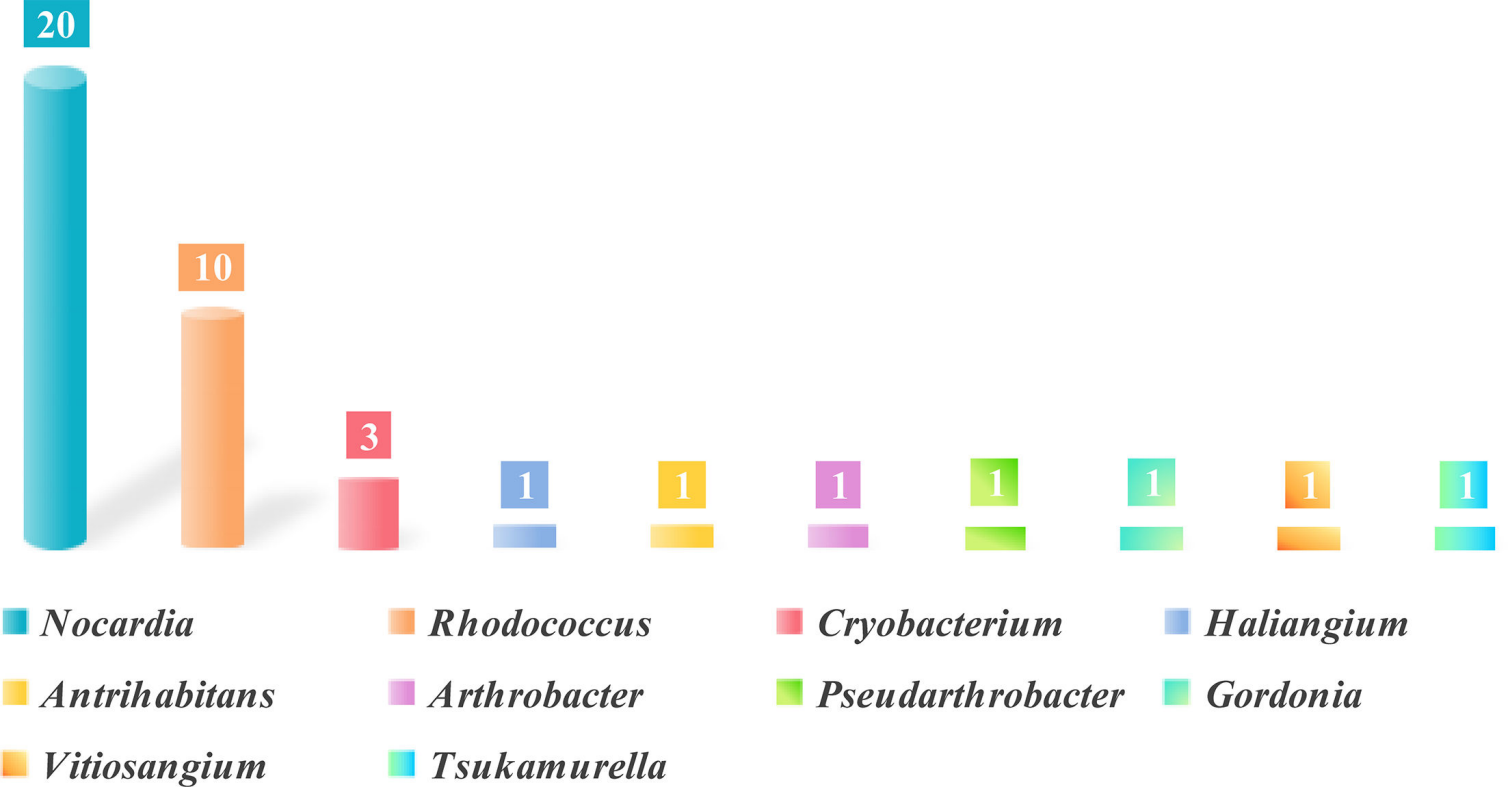
Genus distribution of SkaA homologs in related strains in the RefSeq database (>40% identity).

Homology searches against the NCBI RefSeq database (to date March 2025; 36,754 genomes, 333,218,737 proteins) identified 1,492 putative SkaA homologs (≥30% amino acid identity), of which only 39 exhibited ≥40% identity (Table S7, Fig. S9). These 39 homologs were distributed across 10 actinobacterial genera (*Antrihabitans*, *Arthrobacter*, *Cryobacterium*, *Gordonia*, *Haliangium*, *Nocardia*, *Pseudarthrobacter*, *Rhodococcus*, *Tsukamurella*, and *Vitiosangium*) within the phylum Actinomycetota (Fig. 3). *Nocardia* (20 sequences, 51.3%) and *Rhodococcus* (9 sequences, 23.1%) were the predominant genera. This taxonomic distribution aligned with the established role of Actinomycetota in aerobic aromatic catabolism, particularly via oxygenase-dependent pathways. The prevalence of *Nocardia* and *Rhodococcus*, genera renowned for diverse redox enzyme systems, suggested their potential as key environmental degraders of skatole. While *Rhodococcus* skatole degraders have been recently documented, the role of *Nocardia* warrants investigation. It was notable that SkaA sequences within *R. aetherivorans* exhibited >99% identity, indicating high conservation (24). In contrast, the *Nocardia* homologs displayed lower similarity (40.3–42.0%).

Analysis of the NCBI non-redundant (NR) database (to date April 2025) expanded the data set to 56 homologs (≥40% identity), spanning ten genera across four phyla: Actinomycetota (41, 73.2%), Myxococcota (9, 16.1%), Acidobacteriota (4, 7.1%), and Pseudomonadota (2, 3.6%) (Fig. S10, Table S8). *Nocardia* (15 sequences) and *Rhodococcus* (14 sequences) remained the dominant genera. This broader analysis revealed novel homologs in understudied phyla, particularly Myxococcota.

### SkaA belongs to Group E FPMOs

**Fig 4:**
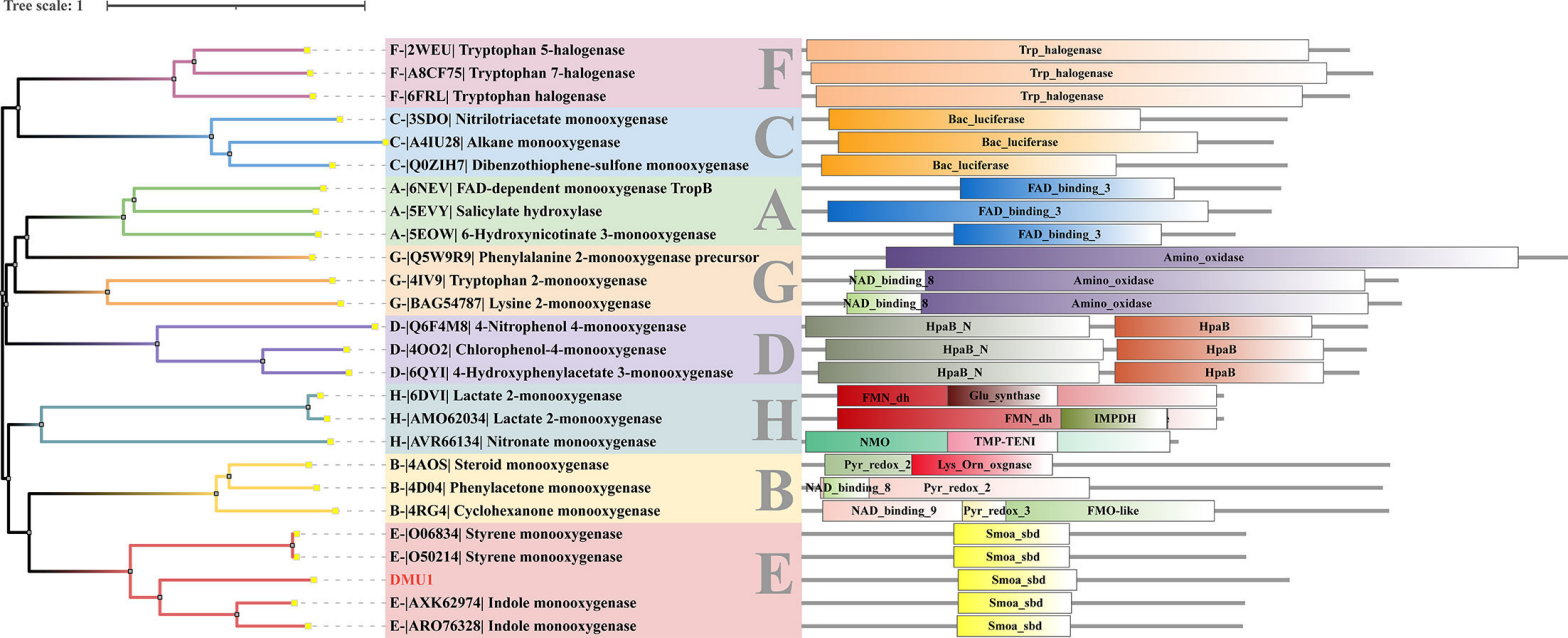
Phylogenetic and domain analyses of different types of FPMOs. Related information is provided in Table S9.

FPMOs represent a versatile class of biocatalysts that mediate chemo-, regio-, and enantioselective oxyfunctionalization reactions across diverse biological processes (30). Based on conserved sequence and structural features, they are categorized into eight distinct phylogenetic groups (Groups A–H). To resolve their evolutionary relationships and domain architectures, we constructed a comprehensive phylogenetic tree using representative FPMO sequences coupled with domain analysis (Fig. 4; Table S9). Group A universally contains the Pfam01494 domain (FAD_binding_3 superfamily cl21454), mediating flavin adenine dinucleotide (FAD) binding (31). Group B, also a member of the cl21454 superfamily, is characterized by the NAD_binding_8 and Pyr_redox_3 domains, which are involved in NAD(P)H binding and pyridine ring oxidation-reduction reactions, respectively. Group C features the luciferase-like Pfam00296 domain, utilizing reduced flavin cofactors (FMNH2/FADH2) for oxygen activation rather than direct flavin substrate binding (32). Group D is characterized by dual domains 4-hydroxyphenylacetate 3-hydroxylase C-terminal HpaB (Pfam03241) and HpaB_N (Pfam11794) in hydroxylation cascades. Group E is defined by conserved styrene monooxygenase domains catalyzing epoxidation. Group F comprises tryptophan halogenases mediating regioselective halogenation. Group G contains Pfam01593 (flavin-dependent amine oxidoreductases) with integrated NAD_binding domains (33). Group H belongs to the cl21457 superfamily and possesses TIM-like beta/alpha barrel domains.

Phylogenetic analysis unequivocally positioned the SkaA within Group E FPMOs (Fig. 4). Enzymes in this group typically operate via a two-component system, where a dedicated flavin reductase supplies reduced FAD to the monooxygenase subunit (34). Based on substrate specificity, Group E FPMOs were primarily divided into styrene monooxygenases (SMOs) and indole monooxygenases (IMOs) (29). Comparative sequence analysis revealed SkaA-DMU1 shared limited homology with established subtypes: 23.8–29.0% identity with SMOs and 27.7–35.1% with IMOs (Table S10). Despite showing closer affinity to IMOs, SkaMOs lacked conserved catalytic motifs characteristic of both subtypes, specifically the SMO fingerprint (N46-V48-H50-Y73-H76-S96) and IMO fingerprint (S46-Q48-M50-V/I73-I76-A96) (Fig. S11) (35).

### SkaMOs form a new branch of Group E FPMOs

**Fig 5:**
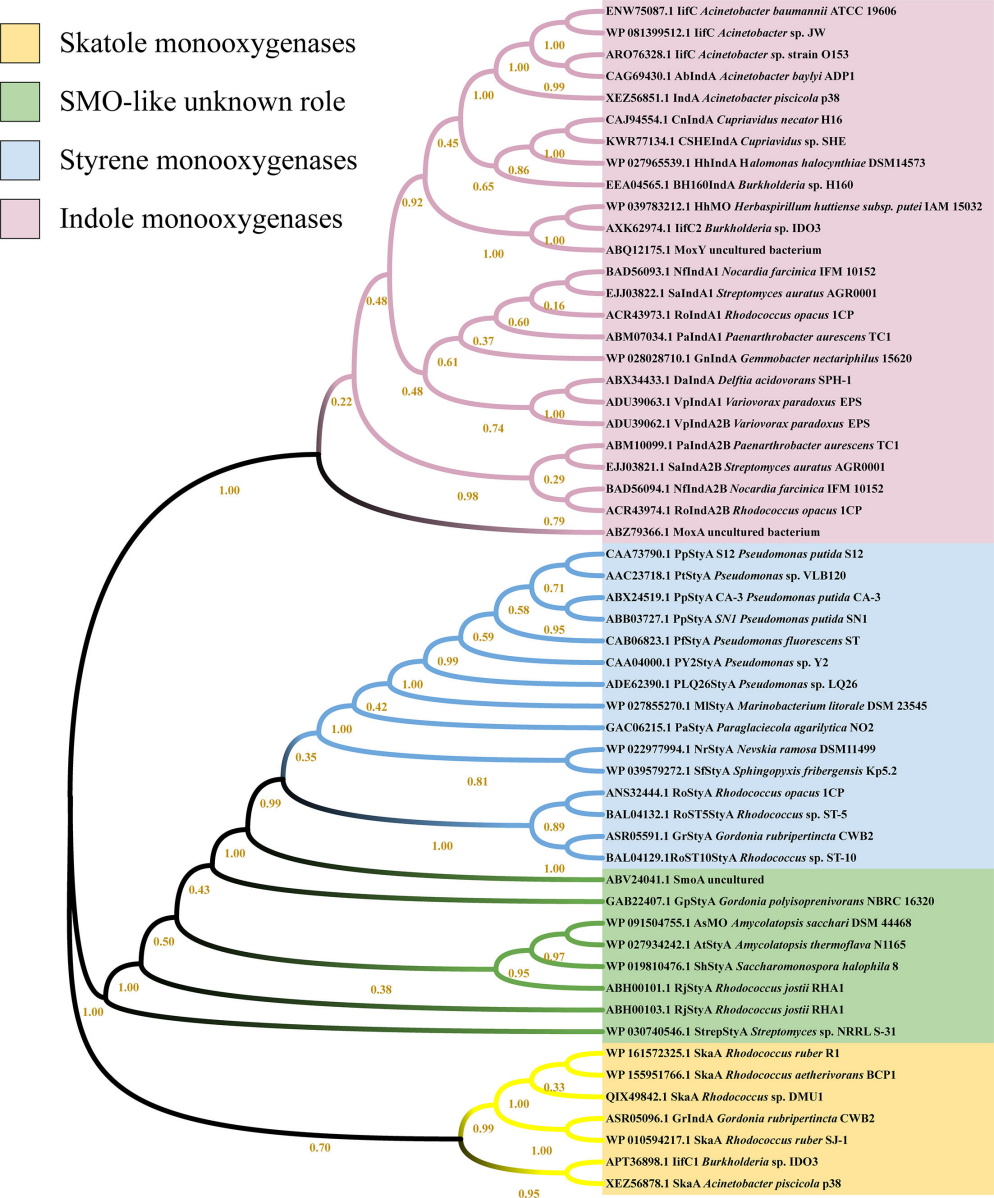
Phylogenetic tree analysis of the reported Group E FPMOs. IMO, indole monooxygenase, in pink. SMO (styrene monooxygenase) with known roles, in blue. SMO-like enzymes with unknown physiological role, in green. SkaMO, skatole monooxygenase, in yellow.

A comprehensive phylogenetic reconstruction including all characterized IMOs and SMOs demonstrated three evolutionarily divergent branches: IMOs, SMOs (including SMO-like enzymes with unknown physiological role), and SkaMOs (Fig. 5). Remarkably, the SkaMOs clade formed a monophyletic cluster comprising both gram-positive (*Rhodococcus* spp.) and gram-negative (*Burkholderia* and *Acinetobacter*) variants (12, 15, 21, 22, 24, 36, 37). This phylogeny enabled functional predictions for several oxygenases: (i) the previously reported *Gordonia rubripertincta* IndA (ASR05096) likely represented a SkaMO homolog (29, 38); (ii) the IifC1 (APT36898) from strain *Burkholderia* sp. IDO3 might be a SkaMO, while IifC2 (AXK62947) was an IMO (36, 39); and (iii) protein XEZ56878 from strain *Acinetobacter piscicola* p38 was a potential SkaMO, while XEZ56851 was an IMO (15). These findings established SkaMOs as a mechanistically distinct subclass within Group E FPMOs, necessitating an expansion of current classification frameworks.

To further validate the function of SkaA homologs in skatole transformation, we heterologously expressed the *skaA* genes from strains SJ-1 (WP_010594217.1, 38.2% similarity) and R1 (WP_161572325.1, 98.9% similarity) (Fig. S12a). The functional activity of the recombinant strains was evaluated by measuring skatole concentration over time in both growing and resting bacterial cell systems (Fig. S12b and c). Both strains could degrade skatole. The strain expressing SJ-1-*skaA* was more effective than the R1-*skaA* strain in degrading skatole under both conditions.

## DISCUSSION

The present study systematically elucidates skatole degradation performance, metabolic pathways, and genetic determinants within the environmentally versatile genus *Rhodococcus*. Our results demonstrate efficient degradation across eight strains and identify a novel monooxygenase, SkaA, which catalyzes the initial oxidation step. Phylogenetic analysis reveals SkaMOs define a distinct subclass within Group E FPMOs, diverging from well-documented IMOs and SMOs. These findings provide mechanistic insights into skatole catabolism in gram-positive bacteria and establish *Rhodococcus* as a promising biocatalyst for skatole remediation.

We first established skatole degradation as a conserved trait within the genus *Rhodococcus*. All eight tested strains, spanning different species, exhibited skatole degradation activity (Fig. S1 and S2; Table 1). This significantly expands prior knowledge where only three species (*R. aetherivorans*, *R. ruber*, and *R. pyridinivorans*) are documented skatole degraders (9, 20, 22–24). Although degradation efficiency is strain- and medium-dependent (e.g., *R. qingshengii* DMU2 achieved complete degradation in 48 h in minimal medium, whereas conspecific strain E7 showed limited activity), the universal capability across phylogenetically distinct isolates underscores *Rhodococcus*’s adaptability as a skatole degrader. This aligns with the genus’s recognized genomic plasticity and redundant aromatic metabolic pathways (40, 41). Our study further identifies *skaA* as a core functional gene conserved in *R. aetherivorans* and *R. ruber* (Fig. 5; Table S7). Strikingly, *skaA*-independent degradation is observed in *R. pyridinivorans* (Rp3, DMU114, and DMU2021), *R. qingshengii* (E7, DMU2), and marine isolates SJ-2/SJ-3, demonstrating alternative genetic pathways and highlighting the genus’s metabolic versatility (42). The underlying mechanisms in these *skaA*-deficient strains remain to be elucidated and may represent another conserved strategy across *Rhodococcus* lineages (43). Collectively, this functional versatility positions *Rhodococcus* as a promising and robust biocatalyst for skatole deodorization, particularly in high-load environments such as livestock manure systems.

We secondly elucidated a novel skatole epoxidation pathway initiated by SkaA, resolving key uncertainties regarding its catabolism. LC-HRMS identified 3-methyloxindole and 3-hydroxy-3-methyloxindole as metabolites in both recombinant *E. coli* and wild-type *R. aetherivorans* DMU1 (Fig. 1). Based on the well-established mechanisms for SMOs and IMOs, which epoxidize styrene and indole to styrene oxide and indole-2,3-epoxide, respectively, we reasonably propose that SkaA catalyzes the formation of 2,3-epoxy-3-methylindole (Fig. S8) (29, 30, 34). This unstable epoxide is expected to spontaneously rearrange to 3-methyl-3H-indol-3-ol and 3-methyloxindole, the latter has been previously detected in anaerobic microbial systems (e.g., methanogenic and sulfate-reducing conditions) and proposed as intermediate in certain *Pseudomonas* and *Acinetobacter* strains (13, 14). Although the conversion of skatole to 3-hydroxy-3-methyloxindole was previously reported in a gram-positive bacterium by Fujioka and Wada (44), the enzymatic basis remained elusive. Our study links SkaA to the initial epoxidation step driving this process. Given that 3-hydroxy-3-methyloxindole is detected in both resting cell and pure enzyme assays, we propose it is generated either through SkaA-mediated hydroxylation of 3-methyloxindole/3-methyl-3H-indol-3-ol or via a spontaneous chemical transformation of the unstable intermediates. However, in analogous indole metabolism, indole-2,3-oxide is catalyzed to 3-hydroxyindolin-2-one by a short-chain dehydrogenase (Fig. S8) (45, 46). Therefore, further research is required to fully elucidate the precise mechanism of 3-hydroxy-3-methyloxindole formation and the subsequent downstream steps in skatole degradation.

Cross-domain similarities in skatole oxidation mechanisms underscore their potential universality. In animal systems, human CYP450 enzymes bioactivate skatole via multiple routes, including epoxidation (to 2,3-epoxyskatole and 3-methyloxindole), hydroxylation (to indole-3-carbinol), and dehydrogenation (to 3-methyleneindolenine) (47). Similarly, pig liver microsomes primarily convert skatole into 3-methyl-3H-indol-3-ol, 3-methyloxindole, and 3-hydroxy-3-methyloxindole (48). This mechanistic parallelism is noteworthy given the widespread presence of CYP450 genes in bacterial genomes, suggesting their potential, yet uncharacterized, role in bacterial skatole detoxification.

We finally identified SkaA as the founding member of a novel subclass within Group E FPMOs (Fig. 5), designated here as SkaMOs. Previous work by Tischler et al. has extensively established that Group E FPMOs are primarily divided into two functional subclasses: SMOs and IMOs, both catalyzing the epoxidation of their respective aromatic substrates (29, 49, 50). Our comprehensive phylogenetic analysis reveals a distinct third branch characterized by skatole catabolism. Evolutionarily, the emergence of specialized FPMOs for these three natural aromatic compounds (styrene, indole, and skatole) suggests a substrate-driven divergence. Within Actinobacteria, SkaMOs appear to exhibit dual conservation patterns: high sequence conservation (>98% identity) within *R. aetherivorans* and *R. ruber*, versus divergent homologs (~40% identity) in *Nocardia* (Tables S8 and S9) (20, 24). This pattern implies lineage-specific adaptation to skatole despite shared catalytic mechanisms. Notably, skatole degradation has not yet been reported in *Nocardia*, identifying a gap for future research. SkaMO homologs identified in gram-negative degraders (*Cupriavidus*, *Pseudomonas*, and *Burkholderia*) show less than 40% identity to *Rhodococcus* variants, indicating convergent evolution of distinct molecular solutions (Table S5) (16, 18).

It is interesting that multiple strains harbor dual oxygenase systems with partitioned substrate specificity in both gram-positive (e.g., *R. ruber* R1 and *R. aetherivorans* BCP1) and gram-negative strains (e.g., *Burkholderia* sp. IDO3 and *Acinetobacter piscicola* p38) (Table S5) (24, 36, 39). Functional studies corroborate this specialization. In *Burkholderia* sp. IDO3, *iifC1* (a putative SkaMO) was significantly upregulated upon skatole stress, and its deletion did not impair indole metabolism, whereas deletion of *iifC2* (a putative IMO) suppressed indole degradation (36). Similarly, in *Acinetobacter piscicola* p38, the *gene1687* (XEZ56878, a putative SkaMO) was significantly upregulated upon skatole stress, while *gene1650* (XEZ56851, a putative IMO) was induced by indole (15). The putative SkaMO in strain p38 could oxidize both skatole and indole, while p38-IMO could only oxidize indole. In this study, strain DMU1 possesses only one *skaA* gene, genetically distinct from those in *R. ruber* R1 and *R. aetherivorans* BCP1. qPCR confirms its involvement in degrading both skatole and indole, indicating complex functional roles based on genomic divergence (Fig. S5). The phylogenetic separation between IMO and SkaMO clades in these strains offers insights into predicting novel skatole-degrading systems in related microorganisms and sheds light on the substrate specificities of uncharacterized Group E FPMOs.

### Conclusion

This study establishes *Rhodococcus* as a versatile genus capable of skatole degradation, revealing previously unrecognized metabolic versatility. We identified a novel skatole epoxidation pathway initiated by the flavin-dependent monooxygenase SkaA. Crucially, phylogenetic analysis redefined Group E FPMOs by establishing SkaMO as a distinct evolutionary clade, exhibiting ≤40% sequence identity to typical styrene/indole monooxygenases and spanning multiple bacterial genera. The discovery of *skaA*-independent degradation pathways further highlights the genus’s adaptive potential. These findings provide fundamental insights for engineering targeted bioremediation strategies in skatole-polluted environments and enrich our understanding of the classification Group E FPMOs.

## MATERIALS AND METHODS

### Chemicals and media

Skatole was purchased from Aladdin (Shanghai, China). The culture media were prepared as follows. Mineral salt (MS) medium (per liter): (NH_4_)_2_SO_4_ 134 mg, KH_2_PO_4_ 141 mg, K_2_HPO_4_ 287 mg, MnSO_4_·H_2_O 2.68 mg, MgSO_4_·7H_2_O 21.4 mg, FeCl_3_·6H_2_O 0.134 mg, and CaCl_2_ 3.8 mg. Mineral salt yeast extract (MSY) medium was prepared from MS medium by adding 1 g/L yeast extract. Luria-Bertani (LB) medium (per liter): tryptone 10 g, NaCl 10 g, and yeast extract 5 g.

### Skatole degradation by *Rhodococcus* strains

*Rhodococcus* strains DMU1, DMU2, DMU114, DMU2021, E7, SJ-1, SJ-2, and SJ-3, which were isolated and preserved in our lab (19, 22), were separately cultured in MSY medium. Upon reaching the logarithmic growth phase, cells were harvested, washed, and resuspended in phosphate-buffered saline (PBS, pH 7.0) to an OD_600_ of 1.0. A 1% (vol/vol) inoculum was then transferred to both MS and MSY media supplemented with 40 mg/L skatole to evaluate degradation capability, with uninoculated media serving as controls. All groups included three biological replicates and were incubated at 30°C with shaking at 150 rpm. Skatole degradation was monitored by sampling at 0, 48, and 96 h, followed by analysis using ultra-performance liquid chromatography (UPLC, Waters) under specified conditions: detection wavelength 280 nm, flow rate 0.8 mL/min, and mobile phase methanol:water (60:40, vol/vol).

### Genome analyses and strain identification

Draft genomes of strains DMU2 and E7 were sequenced using the Illumina platform, and complete genomes of strains SJ-2 and SJ-3 were generated using the PacBio Sequel IIe platform. Genomes for the remaining strains were retrieved from previous studies (22, 26–28, 51). Comparative genomic analysis of all eight *Rhodococcus* strains was performed with BRIG 1.4.1 software, utilizing the complete genome of *R. aetherivorans* DMU1 as the reference. For taxonomic classification, pairwise genome comparisons against 45 validated *Rhodococcus* species were conducted through average nucleotide identity (ANI) analysis using the JSpeciesWS web service (https://jspecies.ribohost.com/jspeciesws/) (52). Digital DNA-DNA hybridization (dDDH) values derived from the d4 formula were calculated via the TYGS platform (https://tygs.dsmz.de/) (53). Taxonomic species were delineated using thresholds of ANI ≥ 95% and dDDH ≥ 70%.

### Quantitative real-time PCR (qPCR) assay

Gene expression was analyzed by quantitative real-time PCR (qPCR). Total RNA was extracted from bacterial cultures using a Vazyme RNA extraction kit and reverse-transcribed into cDNA using the Hiscript III Reverse Transcriptase kit (Vazyme, China). qPCR reactions were performed using ChamQ Universal SYBR qPCR Master Mix (Vazyme, China) on a real-time PCR system (LightCycler 480II, Roche, Switzerland). The 16S rRNA gene was used as the internal reference for normalization. Relative expression level of the target gene was calculated using the 2^−ΔΔCT^ method. All reactions were performed in triplicate, and primers were listed in Table 3.

**TABLE 3:** Strains, plasmids, and primers used in this study

Strain, plasmid, or primer	Genotype, description, or primer sequence (5′ to 3′)	Reference, origin, or description
Strains		
*E. coli* DH5α	Strain used for cloning and plasmid maintenance	Vazyme
*E. coli* BL21(DE3)	Strain used for protein expression	Vazyme
Plasmids		
pET28a(+)	Expression vector; Km^r^	Vazyme
pET32a(+)	Expression vector; Amp^r^	Sangon
pET28a-DMU1-*skaA*	For expression of *skaA* from *Rhodococcus aetherivorans* DMU1; Km^r^	This study
pET28a-SJ-1-*skaA*	For expression of *skaA* from *Rhodococcus ruber* SJ-1; Km^r^	This study
pET32a-R1-*skaA*	For expression of *skaA* from *Rhodococcus ruber* R1; Amp^r^	This study
Primers (5′ to 3′)		
DMU1 16S-F	TTCACACATGCTACAATG	This study
DMU1 16S-R	GCTGATCTGCGATTACTA	This study
*skaA*-qF	AGAAGAATCTCCGTGATC	This study
*skaA*-qR	GTGTAGAGGTCTACTTGG	This study
pET28a-F	CTCGAGCACCACCACCACC	This study
pET28a-R	GGATCCGCGACCCATTTG	This study
DMU1-*skaA*-F	TCATCACCACAGCCAGGATCCGATGAGAAGAATCTCCGTGAT	This study
DMU1-*skaA*-R	GCATTATGCGGCCGCAAGCTTTCACGCGCTCACCGCCCG	This study
SJ-1-*skaA*-F	AGCAAATGGGTCGCGGATCCATGACCACCACTCGACGTTCC	This study
SJ-1-*skaA*-R	TGGTGGTGGTGGTGCTCGAGGGCGGGCTGGACCGCTGC	This study

### Gene expression and function verification

Primers targeting *skaA* from strains DMU1 and SJ-1 (Table 3) were designed to clone the gene into pET-28a(+) expression vector. Gene *skaA* from strain R1 was synthesized by Sangon Biotech (Shanghai, China) and expressed in pET-32a(+) using the *Nco*I and *Xho*I sites. The resulting recombinant plasmid was transformed into *E. coli* DH5α for sequence verification before electroporation into *E. coli* BL21(DE3) for functional studies. Transformants were cultivated in Luria-Bertani (LB) medium with 100 mg/L kanamycin or 50 mg/L ampicillin at 37°C and 150 rpm. For heterologous expression, the recombinant strain was induced at an OD_600_ of 0.4 by adding 0.2 mM isopropyl-β-d-thiogalactoside and further incubated at 16°C overnight. For resting cell assays, harvested cells were washed and resuspended in PBS. Reactions were initiated by adding 50 mg/L skatole (treatment), followed by incubation at 30°C with shaking. For pure enzyme assays, recombinant cells were lysed and the His-tagged protein was purified using a Ni-NTA column (22). The enzymatic reaction mixture consisted of 20 μM FAD, 2 mM NADH, a specified amount of the purified enzyme, and skatole. Reactions were carried out at 30°C with shaking for 24 h.

### Metabolite identification

The transformation metabolites of skatole generated by strains DMU1 and *E. coli* expressing DMU1-*skaA* were analyzed by liquid chromatography–high-resolution mass spectrometry (LC-HRMS). Analyses were performed on a Thermo Fisher Q Exactive Plus Orbitrap mass spectrometer equipped with an electrospray ionization source. The spray voltage was 3.6 kV, capillary temperature 320°C, ion source temperature 320°C, sheath gas flow 35, auxiliary gas flow 10, and probe heater (desolvation line) temperature 450°C. Chromatographic separation was carried out on a Thermo Scientific Hypersil GOLD C18 column (100 × 2.1 mm, 1.9 µm) using the mobile phase of 0.1% formic acid in water (A) and acetonitrile (B). A gradient elution was applied as follows: 0–2 min, 5% B; 2–13 min, 5–100% B; 13–16 min, 100% B; 16–20 min, 5% B, at a flow rate 0.2 mL/min, and column temperature 40°C.

### Distribution analysis of SkaA homologs

To investigate the distribution of skatole oxygenase homologs, we screened two microbial proteome databases constructed from NCBI resources: the RefSeq database (333,218,737 predicted protein sequences, March 2025) and the non-redundant (NR) database (707,028,945 predicted protein sequences, April 2025) (54). Using DIAMOND v2.1.11 with uniform parameters (sequence identity >40%, query/subject coverage >70%), we queried both databases against the reference skatole oxygenase SkaA (QIX49842.1) from *R. aetherivorans* DMU1. Homologous sequences were taxonomically classified to determine phylum- and genus-level distribution patterns. For phylogenetic reconstruction, candidate homologs were aligned with MAFFT, and a maximum-likelihood tree was constructed with FastTree v2.1.11. Evolutionary relationships were visualized and annotated in iTOL.

### Analysis of the *ska* gene cluster in skatole-degrading strains

The SkaA protein sequence from strain DMU1 served as the reference for BLASTP analysis (*E* value ≤ 1 × 10⁻⁵) against all available genomes of reported skatole-degrading bacteria. Matching sequences were mapped to corresponding coding sequences in GenBank annotations to identify potential gene clusters. Gene cluster visualizations were generated using ChiPlot (https://www.chiplot.online/).

### Phylogenetic analysis of skatole, indole, and styrene monooxygenases

Phylogenetic analysis of the skatole, indole, and styrene monooxygenases was conducted by aligning the protein sequences using ClustalW. The treated sequences were used to construct a maximum likelihood phylogenetic tree (Jones-Taylor-Thornton amino acid substitution model) with 1,000 bootstrap replicates by MEGA12 (29).

## Data Availability

The genomic sequencing data from this study have been deposited to NCBI under the BioProject accession numbers PRJNA1295803 (DMU2), PRJNA1295815 (E7), PRJNA1295737 (SJ-2), and PRJNA1301114 (SJ-3).

## SUPPLEMENTAL MATERIAL

Supplemental material aem.00228-26-s0001.pdf Tables S1 to S10; Fig. S1 to S12.

ASM does not own the copyrights to Supplemental Material that may be linked to, or accessed through, an article. The authors have granted ASM a non-exclusive, world-wide license to publish the Supplemental Material files. Please contact the corresponding author directly for reuse.
