# Supplementary material for: A novel and evolutionarily distinct flavoprotein monooxygenase drives skatole degradation in Rhodococcus
Source: Appl Environ Microbiol. 2026 Mar 25;92(4):e00228-26. doi: 10.1128/aem.00228-26 (PMC13101512; doi:10.1128/aem.00228-26)
Supplement: Supplemental material — Tables S1 to S10; Fig. S1 to S12. [file aem.00228-26-s0001.pdf]

Supplementary materials for

A novel and evolutionarily distinct flavoprotein monooxygenase drives skatole degradation in *Rhodococcus*

Nan Meng, Genxiu Li, Jiaxin Zhang, Jingwen Zhu, Xutong Kang, Jiahao Duan, Qiao Ma*

Institute of Environmental Systems Biology, College of Environmental Science and Engineering, Dalian Maritime University, Dalian 116026, China

*Corresponding author: Qiao Ma, e-*mail*: xiaoma0556@dlmu.edu.cn, College of Environmental Science and Engineering, Dalian Maritime University, Dalian 116026, China

Tables	10
Figures	12

Table S1 Summary of skatole-degrading strains

Strain	Degradation ability	Reference
	**Gram-negative strains**	
Pseudomonas aeruginosa Gs	Degrade 2 mM skatole within 24 days	(1)
Pseudomonas putida LPC24	Degrade 2 mM skatole within 30 days	(2)
Acinetobacter strains NTA1-2A and TAT1-6A	Degrade > 85% skatole (< 200 mg/L) in 6 days	(3)
Acinetobacter oleivorans AO-06	Degrade skatole (< 300 mg/L) within 120 h	(4)
Acinetobacter xiamenensis Ya	Degrade 80 mg/L skatole within 72 h	(5)
Acinetobacter piscicola p38	Exploration of potential degradation genes	(6)
Burkholderia sp. IDO3	Degrade 50 mg/L skatole within 24 h	(7)
Sulfate-reducing bacteria	Degrade 2 mM skatole within 4 weeks	(8)
Cupriavidus sp. KK10	Degrade 100 mg/L skatole within 24 h	(9)
Rhodopseudomonas palustris WKU-KDNS3	Remove > 90% skatole (0.1 mM) in 21 days	(10)
	**Gram-positive strains**	
Unidentified Gram-positive bacterium	The removal rate is 32% in 4 weeks (100–300 mg/L skatole)	(11)
Lactobacillus brevis 1.12	The highest removal rate is 65% in 120 h (1.0 mg/L skatole)	(12)
Rhodococcus aetherivorans DMU1	Degrade 50 mg/L skatole within 24 h	(13)
Rhodococcus pyridinivorans Rp3	Degrade 100 mg/L skatole within 48 h	(14)
Rhodococcus ruber R1	Degrade 1 mM skatole within 30 h	(15)
Rhodococcus aetherivorans BCP1	Degrade 0.5 mM skatole within 48 h	(15)
Bacillus subtilis GDAAS-A32	Degrade 100 mg/L skatole within 40 h	(16)

Reference

1. Yin B, Huang L, Gu J-D. 2006. Biodegradation of 1-methylindole and 3-methylindole by mangrove sediment enrichment cultures and a pure culture of an isolated *Pseudomonas aeruginosa* Gs. *Water, Air, and Soil Pollution* 176: 185–199.
2. Li P, Tong L, Liu K, Wang Y. 2010. Biodegradation of 3-methylindole by *Pseudomonas putida* LPC24 under oxygen limited conditions. *Fresenius Environmental Bulletin* 19: 238–242.
3. Tesso TA, Zheng A, Cai H, Liu G. 2019. Isolation and characterization of two *Acinetobacter* species able to degrade 3-methylindole. *PLoS ONE* 14: e0211275.
4. Hu H, Li L, Gao F, Diao W, Ma H, Feng F, Quan S, Xiang L, Zhang X. 2022. Screening, identification, and degradation characteristics of 3-methylindole degrading bacteria. *Frontiers in Environmental Science* 10: 1028699.
5. Ye J, Fu Q. 2023. Screening of skatole-degrading bacteria and control of human fecal odor by compound bacteria. *Annals of Microbiology* 73: 22.
6. Wang Z, Sun J, Yang P, Zhang W, Jiang Y, Liu Q, Yang Y, Hao R, Guo G, Huo W, Zhang Q, Li Q. 2024. Molecular analysis of indole and skatole decomposition metabolism in *Acinetobacter piscicola*p 38 utilizing biochemical and omics approaches. *Microorganisms* 12: 1792.
7. Ma Q, Qu H, Meng N, Li S, Wang J, Liu S, Qu Y, Sun Y. 2020. Biodegradation of skatole by

Burkholderia sp. IDO3 and its successful bioaugmentation in activated sludge systems. *Environmental Research* 182: 109123.

8. Bak F, Widdel F. 1986. Anaerobic degradation of indolic compounds by sulfate-reducing enrichment cultures, and description of *Desulfobacterium indolicum*gen. nov., sp. nov. *Archives of Microbiology* 146: 170–176.
9. Fukuoka K, Ozeki Y, Kanaly RA. 2015. Aerobic biotransformation of 3-methylindole to ring cleavage products by *Cupriavidus* sp. strain KK10. *Biodegradation* 26: 359–373.
10. Sharma N, Doerner KC, Alok PC, Choudhary M. 2015. Skatole remediation potential of *Rhodopseudomonas palustris* WKU-KDNS3 isolated from an animal waste lagoon. *Letters in Applied Microbiology* 60: 298–306.
11. Kohda C, Ando T, Nakai Y. 1997. Isolation and characterization of anaerobic indole- and skatole-degrading bacteria from composting animal wastes. *Journal of General and Applied Microbiology* 43: 249–255.
12. Meng X, He ZF, Li HJ. 2013. Purification and characterization of a novel skatole-degrading protease from *Lactobacillus brevis*1.12. *Food Science and Biotechnology* 22: 1–7.
13. Ma Q, Liu S, Li S, Hu J, Tang M, Sun Y. 2020. Removal of malodorant skatole by two enriched microbial consortia: Performance, dynamic, function prediction and bacteria isolation. *Science of the Total Environment* 725: 138416.
14. Wu Y, Zhang S, Tian Q, Ma G, Guo R, Li S, Zhong Z. 2021. Isolation and identification of a high-efficiency bacterial strain Rp3 to degrade skatole: An odor chemical in compost. *Journal of Agricultural Resources and Environment* 38: 576–584.
15. Galaz SJ, Saavedra B, Zúñiga A, González-Toro F, Donoso R. 2024. Aniline dioxygenase in *Rhodococcus ruber* R1: Insights into skatole degradation. bioRxiv. https://doi.org/10.1101/2024.12.29.630347.
16. Hu J-Y, Han X-Y, Chen H-P, Li J-Z, Wang Z-L, Luo X-C, Deng J-J. 2025. Biodegradation of skatole by *Bacillus subtilis* GDAAS-A32 and its inhibition for odor emissions from swine manure. *Journal of Environmental Chemical Engineering* 13: 115426.

Table S2 ANI values between the *Rhodococcus* strains

**Strain**	**DMU1**	**DMU2**	**DMU114**	**DMU2021**	**E7**	**SJ-1**	**SJ-2**	**SJ-3**
**DMU1**	100	72.9	78.34	77.99	72.75	91.7	76.26	75.89
**DMU2**	72.71	100	72.31	72	98.59	72.6	71.19	71.06
**DMU114**	77.09	71.67	100	97.54	71.75	77.65	77.3	76.57
**DMU2021**	77.03	71.63	98.11	100	71.69	78.06	77.71	76.67
**E7**	72.75	98.48	72.32	72	100	72.57	71.11	71.07
**SJ-1**	90.18	72.24	77.92	78.26	72.08	100	76.44	75.13
**SJ-2**	75.88	71.06	77.8	78.21	70.89	76.93	100	98.41
**SJ-3**	75.72	71.01	77.18	77	70.89	75.57	98.54	100
R. aetherivorans JCM 14343	97.19	72.3	77.3	77.35	72.1	90.79	75.64	75.27
R. antarcticus 75	71.54	69.62	71.28	71.19	69.6	71.7	69.83	69.73
R. artemisiae YIM 65754	76.34	71.23	77.52	77.42	71.17	76.19	87.01	87.19
R. baikonurensis JCM 11411	72.71	93.53	72.33	72.09	93.65	72.47	71.06	70.93
R. cerastii strain IEGM 1327	72.32	71.52	71.69	71.35	71.29	72.09	70.61	70.63
R. cercidiphylli IEGM 1322	72.01	71.46	71.7	71.45	71.2	72.07	70.53	70.51
R. chondri CC-R104	77.7	71.72	78.16	78.24	71.59	77.55	77.38	77.94
R. coprophilus NCTC10994	76.34	71.41	78.17	77.92	71.32	76.27	75.97	75.87
R. daqingensis CGMCC 1.13630	74.73	72.75	73.80	73.56	72.72	74.72	72.3	72.15
R. erythropolis R138	72.63	94.88	72.25	71.86	95.15	72.51	71.01	71.02
R. fascians DSM 43673	71.92	71.28	71.58	71.34	71.04	71.86	70.51	70.53
R. gannanensis CGMCC 1.15992	74.47	71.98	73.77	73.52	72.02	74.42	72.54	72.32
R. globerulus NBRC 14531	72.34	79.90	71.76	71.53	79.94	72.08	70.82	70.82
R. gordoniae NCTC13296	76.95	71.5	90.01	89.87	71.55	77.01	76.64	76.55
R. indonesiensis SARSHI1	90.15	72.17	77.14	77.03	72.01	94.77	75.51	75.12
R. jostii DSM 44719	75.25	73.86	73.74	73.69	73.78	74.86	72.57	72.34
R. koreensis JCM 10743	75.27	73.76	74	73.75	73.82	75	72.52	72.29
R. kroppenstedtii DSM 44908	72.79	70.92	72.24	72.02	70.79	72.55	70.76	70.76
R. kyotonense JCM 23211	72.02	71.27	71.76	71.55	71.18	72.04	70.44	70.42
R. maanshanensis NBRC 100610	74.67	72.93	73.83	73.56	72.82	74.65	72.33	72.18
R. marinonascens NBRC 14363	73.39	72.86	72.48	72.01	72.71	73.06	71.32	71.17
R. navarretei EXRC-4A-4	72.13	71.24	71.64	71.3	71.11	71.96	70.61	70.51
R. olei JCM 32206	75.14	72.53	74.3	73.99	72.44	75.09	72.69	72.56
R. opacus ATCC 51881	75.23	73.94	73.96	73.86	73.82	74.93	72.4	72.31
R. oryzae NEAU-CX67	74.55	72.78	73.73	73.47	72.78	74.58	72.2	72.03
R. oxybenzonivorans S2-17	74.41	73.17	73.11	72.93	73.15	73.89	71.99	71.69
R. parequi PAM 2766	74.92	72.74	74.28	73.99	72.71	75.06	72.75	72.72
R. pseudokoreensis R79	75.41	73.77	74.09	73.89	73.84	75.08	72.58	72.33
R. pyridinivorans DSM 44555	77.09	71.65	97.92	98.4	71.72	77.31	77.02	76.66

R. qingshengii JCM 15477	72.64	98.35	72.41	71.93	98.5	72.52	71.07	70.99
R. rhodnii ATCC 35071	73.31	71.26	72.95	72.71	71.24	73.13	71.44	71.47
R. rhodochrous NCTC10210	77.01	71.68	93.77	93.24	71.56	77.14	76.71	76.54
R. ruber DSM 43338	90.29	72.19	77.3	77.05	72.13	98.76	75.25	75.11
R. sacchari Z13	77.52	71.78	82.98	82.74	71.82	77.39	76.45	76.33
R. sovatensis 18930	71.48	70.79	71.02	70.78	70.8	71.33	69.9	69.95
R. spelaei C9-5	74.73	72.98	73.96	73.69	72.97	74.72	72.43	72.32
R. spongiicola LHW50502	74.19	72.23	73.37	73.24	72.18	74.01	71.96	72.11
R. triatomae DSM 44893	74.13	72.33	73.53	73.19	72.26	74.01	71.97	71.96
R. trifolii CCM 7905	71.66	70.76	71.3	71	70.66	71.59	70.14	70.19
R. tukisamuensis JCM 11308	74.92	73.7	74.63	74.19	73.58	75.42	72.79	72.77
R. wratislaviensis YZ02	75.37	73.93	73.86	73.69	73.86	74.95	72.44	72.24
R. xishaensis LHW51113	74.2	72.17	73.42	73.22	72.02	73.89	72	72.05
R. yananensis FBM22-1	76.74	71.47	77.57	77.29	71.51	76.69	77.35	77.25
R. yunnanensis NBRC 103083	71.71	71.03	71.4	71.1	70.99	71.67	70.16	70.19
R. zopfii NBRC 100606	78.75	71.97	77.66	77.97	71.88	78.49	76.23	76.07

Table S3 dDDH values between the *Rhodococcus* strains

Query strain	Subject strain	dDDH (d4, in %)	G+C content difference (in %)
DMU1	*R. aetherivorans* JCM 14343	91.4	0.24
DMU1	*R. aetherivorans* DSM 44752	91.3	0.23
DMU2	E7	89.9	0
DMU2	*R. jialingiae* djl-6-2	89.1	0.08
DMU2	*R. qingshengii* JCM 15477	88.5	0.13
DMU2	*R. enclensis* DSM 45688	87.9	0.19
DMU114	*R. pyridinivorans* DSM 44555	85.7	0.03
DMU114	DMU2021	86.2	0.22
DMU2021	*R. pyridinivorans* DSM 44555	91.1	0.19
DMU2021	*R. biphenylivorans* TG9	88.4	0.39
E7	*R. enclensis* DSM 45688	89.9	0.19
E7	*R. qingshengii* JCM 15477	89.5	0.13
SJ-1	*R. ruber* NBRC 15591	92.9	0.32
SJ-1	*R. ruber* DSM 43338	92.7	0.3
SJ-2	SJ-3	91.6	0.02
SJ-2	*R. artemisiae* YIM 65754	33.9	0.24
SJ-3	*R. artemisiae* YIM 65754	34.1	0.22

Table S4 Potential SkaA homologs (>30% similarity) in all the 53 *Rhodococcus* strains from Table S2

Strain	Subject ID	Percent identity (%)	Query coverage (%)	E-value
Rhodococcus aetherivorans DMU1	QIX49842	100	-	-
Rhodococcus aetherivorans JCM 14343	WP_152103792.1	99.3	100	0
Rhodococcus aetherivorans JCM 14343	WP_043796862.1	38.1	80.7	3.35e-67
Rhodococcus indonesiensis SARSHI1	WP_416062207.1	38.4	80.7	2.82e-69
Rhodococcus opacus ATCC 51881	WP_005563102.1	37.9	80.7	6.01e-65
Rhodococcus opacus ATCC 51881	WP_005568880.1	33.4	91.4	2.73e-65
*Rhodococcus opacu*s ATCC 51881	WP_005568881.1	30.7	91.4	4.68e-53
Rhodococcus oxybenzonivorans S2-17	WP_109335618.1	30.0	92.1	1.17e-30
Rhodococcus ruber SJ-1	WP_010594217.1	38.2	80.7	1.23e-66
Rhodococcus sacchari Z13	WP_264154094.1	36.9	92.3	3.32e-65
Rhodococcus spongiicola LHW 50502	WP_127947546.1	34.0	91.4	5.20e-65
Rhodococcus spongiicola LHW 50502	WP_127947545.1	31.9	91.7	3.31e-55
Rhodococcus triatomae DSM 44893	WP_072736178.1	36.6	80.7	1.57e-60

Table S5 Distribution of potential SkaA homologs (>30% similarity) in all available genomes of skatole-degrading strains

**Strain**	**Protein ID**	**Start**	**End**	**Gene length**	**Similarity**
Rhodococcus aetherivorans DMU1	QIX49842	2091562	2092929	1368	100%
Rhodococcus ruber SJ-1	WP_010594217	3286923	3288296	1374	38.2%
Rhodococcus ruber R1	WP_161572325	1670408	1671775	1368	98.9%
Rhodococcus ruber R1	QDC16752.1	5180175	5181548	1374	38.2%
Rhodococcus aetherivorans BCP1	WP_155951766	1292779	1294146	1368	99.1%
Rhodococcus aetherivorans BCP1	WP_006943660	5068421	5069794	1374	38.2%
Cupriavidus sp. KK10	WP_008642920	6373548	6374789	1242	34.8%
Cupriavidus sp. KK10	WP_013953986	3671115	3672404	1290	32.8%
Burkholderia sp. IDO3	APT36898	820804	822069	1266	35.7%
Burkholderia sp. IDO3	AXK62974	2226214	2227458	1245	32.2%
Acinetobacter piscicola p38	XEZ56851	1690588	1691826	1239	31.6%
Acinetobacter piscicola p38	XEZ56878	1723205	1724476	1272	36.9%
Rhodococcus qingshengii DMU2	-	-	-	-	-
Rhodococcus pyridinivorans DMU114	-	-	-	-	-
Rhodococcus pyridinivorans DMU2021	-	-	-	-	-
Rhodococcus qingshengii E7	-	-	-	-	-
Rhodococcus sp. SJ-2	-	-	-	-	-
Rhodococcus sp. SJ-3	-	-	-	-	-
Rhodococcus pyridinivorans Rp3	-	-	-	-	-

Table S6 The gene cluster responsible for skatole degradation in strain DMU1

Gene	Protein ID	Name	Homologous protein
HFP48_09850	QIX49835.1	SkaC	Aromatic-ring-hydroxylating dioxygenase subunit alpha
HFP48_09855	QIX49836.1	SkaD	Aromatic-ring-hydroxylating dioxygenase subunit beta
HFP48_09860	QIX49837.1	SkaZ	Unknown
HFP48_09865	QIX49838.1	SkaE	Type 1 glutamine amidotransferase
HFP48_09870	QIX49839.1	SkaF	NAD(P)/FAD-dependent oxidoreductase
HFP48_09875	QIX49840.1	SkaG	4Fe-4S binding protein
HFP48_09880	QIX49841.1	SkaH	Glutamine synthetase
HFP48_09885	QIX49842.1	SkaA	Styrene monooxygenase StyA
HFP48_09890	QIX49843.1	SkaI	Ectoine hydrolase
HFP48_09895	QIX49843.1	SkaJ	Hydantoinase subunit B
HFP48_09900	QIX49845.1	SkaK	Hydantoinase subunit A
HFP48_09905	QIX49846.1	SkaR	LuxR C-terminal-related transcriptional regulator
HFP48_09910	QIX49847.1	SkaL	SDR family oxidoreductase
HFP48_09915	QIX49848.1	SkaB	Flavin reductase family protein

Table S7 SkaA homologs in the NCBI RefSeq database with ≥40% identity

ID	Similarity	Genus	Species	Strain
WP_149433161.1	40.7	*Antrihabitans*	*cavernicola*	
WP_237821021.1	58.7	*Arthrobacter*	*hankyongi*	
WP_283501880.1	63.3	*Cryobacterium*	sp.	PH31-O1
WP_283497437.1	62.4	*Cryobacterium*	sp.	PH29-G1
WP_134502114.1	63.7	*Cryobacterium*	*glaciale*	
WP_170194208.1	42.3	*Gordonia*	*asplenii*	
WP_045119590.1	50.5	*Haliangium*	*ochraceum*	
WP_402645705.1	40.9	*Nocardia*	sp.	NPDC 087230
WP_396916410.1	40.6	*Nocardia*	*brasiliensis*	
WP_393442621.1	40.1	*Nocardia*	sp.	NPDC 049149
WP_387700286.1	40.9	*Nocardia*	*thailandica*	
WP_379315658.1	40.9	*Nocardia*	sp.	NPDC 060220
WP_357240208.1	40.9	*Nocardia*	sp.	NPDC 019395
WP_345494243.1	42	*Nocardia*	*callitridis*	
WP_342214927.1	41.3	*Nocardia*	*cyriacigeorgica*	
WP_280441622.1	40.6	*Nocardia*	*brasiliensis*	
WP_280430607.1	40.3	*Nocardia*	*brasiliensis*	
WP_280409824.1	40.9	*Nocardia*	*brasiliensis*	
WP_280404053.1	40.6	*Nocardia*	*brasiliensis*	
WP_280393585.1	40.6	*Nocardia*	*brasiliensis*	
WP_195137510.1	40.6	*Nocardia*	*brasiliensis*	
WP_195091645.1	40.6	*Nocardia*	*brasiliensis*	
WP_043663825.1	40.6	*Nocardia*	*vulneris*	
WP_043651352.1	41.1	*Nocardia*	*thailandica*	
WP_042258574.1	40.3	*Nocardia*	*brasiliensis*	
WP_029900882.1	40.6	*Nocardia*	*brasiliensis*	
WP_014988896.1	40.6	*Nocardia*	*brasiliensis*	
WP_252568485.1	58.3	*Pseudarthrobacter*	sp.	HLT3-5
WP_263507466.1	99.3	*Rhodococcus*	*aetherivorans*	
WP_203329394.1	99.6	*Rhodococcus*		
WP_185113676.1	99.8	*Rhodococcus*	*aetherivorans*	
WP_170914061.1	99.6	*Rhodococcus*		
WP_168579423.1	100	*Rhodococcus*	*aetherivorans*	DMU1
WP_161995899.1	99.6	*Rhodococcus*	sp.	YH1
WP_161572325.1	98.9	*Rhodococcus*	*ruber*	
WP_155951766.1	99.1	*Rhodococcus*	*aetherivorans*	
WP_152103792.1	99.3	*Rhodococcus*	*aetherivorans*	
WP_146034277.1	99.6	*Rhodococcus*		
WP_146560306.1	40	*Tsukamurella*	*asaccharolytica*	
WP_108076155.1	40.1	*Vitiosangium*	sp.	GDMCC 1.1324

Table S8 SkaA homologs in the NCBI NR database with ≥40% identity

ID	Similarity	Genus	Species	Strain
WP_283501880.1	63.3	*Cryobacterium*	sp.	PH
WP_283497437.1	62.4	*Cryobacterium*	sp.	PH
WP_280441622.1	40.6	*Nocardia*	*brasiliensis*	
WP_280430607.1	40.3	*Nocardia*	*brasiliensis*	
WP_280409824.1	40.9	*Nocardia*	*brasiliensis*	
WP_280404053.1	40.6	*Nocardia*	*brasiliensis*	
WP_280393585.1	40.6	*Nocardia*	*brasiliensis*	
WP_280230640.1	40.4	*Nocardia*	*cyriacigeorgica*	
WP_280206591.1	40.2	*Nocardia*	*cyriacigeorgica*	
WP_263507466.1	99.3	*Rhodococcus*	*aetherivorans*	
WP_252568485.1	58.3	*Pseudarthrobacter*	sp.	HLT
WP_237821021.1	58.7	*Arthrobacter*	*hankyongi*	
WP_203329394.1	99.6	*Rhodococcus*		
WP_195137510.1	40.6	*Nocardia*	*brasiliensis*	
WP_195091645.1	40.6	*Nocardia*	*brasiliensis*	
WP_185113676.1	99.8	*Rhodococcus*	*aetherivorans*	
WP_170914061.1	99.6	*Rhodococcus*	sp.	M
WP_170194208.1	42.3	*Gordonia*	*asplenii*	
WP_168579423.1	100	*Rhodococcus*	sp.	DMU1
WP_161572325.1	98.9	*Rhodococcus*	*ruber*	
WP_155951766.1	99.1	*Rhodococcus*	*aetherivorans*	
WP_152103792.1	99.3	*Rhodococcus*	*aetherivorans*	
WP_149433161.1	40.7	*Spelaeibacter*	*cavernicola*	
WP_146560306.1	40	*Tsukamurella*	*asaccharolytica*	
WP_146034277.1	99.6	*Rhodococcus*	*aetherivorans*	
WP_134502114.1	63.7	*Cryobacterium*	*glaciale*	
WP_108076155.1	40.1	*Vitiosangium*	sp.	GDMCC
WP_045119590.1	50.5	*Haliangium*	*ochraceum*	
WP_043663825.1	40.6	*Nocardia*	*vulneris*	
WP_043651352.1	41.1	*Nocardia*	*thailandica*	
WP_042258574.1	40.3	*Nocardia*	*brasiliensis*	
WP_029900882.1	40.6	*Nocardia*	*brasiliensis*	
WP_014988896.1	40.6	*Nocardia*	*brasiliensis*	
OLL18304.1	99.3	*Rhodococcus*	sp.	M8
NCL75270.1	99.6	*Rhodococcus*	sp.	YH
MDQ1502227.1	57.1	unknown	bacterium	
MCU1347542.1	41.8	unknown	bacterium	
MCI0543486.1	52.6	unknown	bacterium	
MCI0425126.1	52.3	unknown	bacterium	
MCI0408171.1	40.9	unknown	bacterium	
MCH8128790.1	47.3	unknown	bacterium	

MCH8056365.1	40.8	unknown	bacterium	
MCH7845050.1	46.7	unknown	bacterium	
KDE14434.1	98.9	*Rhodococcus*	*aetherivorans*	
HYV43985.1	40.2	*Myxococcaceae*	bacterium	
HWN71841.1	46.9	*Haliangium*	sp	
HVV87497.1	41	*Kofleriaceae*	bacterium	
HVR78000.1	47.4	*Acidimicrobiia*	bacterium	
HVE81196.1	40.2	*Myxococcales*	bacterium	
HUF96357.1	48.8	*Acidimicrobiia*	bacterium	
HMV69958.1	44.1	unknown	bacterium	
HEX2573964.1	40	*Polyangia*	bacterium	
GES35234.1	99.1	*Rhodococcus*	*aetherivorans*	
CCW14454.1	99.6	*Rhodococcus*	*aetherivorans*	
ASF11060.2	40.2	*Nocardia*	*brasiliensis*	
ANZ25892.1	99.3	*Rhodococcus*	sp.	WB1
ACY18693.1	50.8	*Haliangium*	*ochraceum*	

Table S9 Flavoprotein monooxygenases used in this study

Group	Annotation	Name	Resource strain
Group A	6-Hydroxynicotinate 3-monooxygenase 6HNMO	PDB 5EOW	*Pseudomonas putida* KT2440
Group A	*p*-Nitrophenol 4-monooxygenase PnpA	6AIN_A	*Pseudomonas putida*
Group A	FAD-dependent monooxygenase tropB	6NEV_A	*Talaromyces stipitatus* ATCC 10500
Group B	Phenylacetone monooxygenase	4D04_A	*Thermobifida fusca*
Group B	Cyclohexanone monooxygenase	4RG4_A	*Rhodococcus* sp. HI-31
Group B	Steroid monooxygenase	4AOS_A	*Rhodococcus rhodochrous*
Group C	Dibenzothiophene-sulfone monooxygenase	Q0ZIH7	*Rhodococcus erythropolis*
Group C	Nitrilotriacetate monooxygenase	3SDO_A	*Burkholderia pseudomallei*
Group C	Long-chain alkane monooxygenase	A4IU28	*Geobacillus thermodenitrificans*
Group D	4-Nitrophenol monooxygenase	UniProtKB Q6F4M8	*Rhodococcus opacus*
Group D	4-Hydroxyphenylacetate 3-monooxygenase oxygenase	6QYI_A	*Escherichia coli*
Group D	Chlorophenol-4-monooxygenase	4OO2_A	*Streptomyces globisporus*
Group E	Styrene monooxygenase	UniProtKB O50214	*Pseudomonas* sp. VLB120
Group E	Styrene monooxygenase	O06834	*Pseudomonas fluorescens* ST
Group E	Indole monooxygenase IifC	ARO76328.1	*Acinetobacter* sp. O153
Group E	Indole monooxygenase IifC	AXK62974	*Burkholderia* sp. IDO3
Group F	Tryptophan 7-halogenase KtzQ	A8CF75	*Kutzneria* sp. 744
Group F	Tryptophan halogenase	6FRL_A	*Brevundimonas* sp. BAL3.
Group F	Tryptophan 5-halogenase (PyrH)	2WEU_A	*Streptomyces rugosporus*
Group G	Lysine 2-monooxygenase LMO	BAG54787	*Pseudomonas putida*
Group G	L-phenylalanine oxidase	Q5W9R9	*Pseudomonas* sp. P-501

Group G	Tryptophan 2-monooxygenase	4IV9_A	*Pseudomonas savastanoi*
Group H	Lactate 2-monooxygenase	AMO62034	*Mycolicibacterium phle*i
Group H	Nitronate monooxygenase	AVR66134	*Pseudomonas paraeruginosa*
Group H	Nitronate monooxygenase	6BKA_A	*Cyberlindnera mrakii*

Table S10 Comparison of SkaA with reported styrene and indole monooxygenases. The colors in the table match those in Fig. 5.

**Description**	**Identity (%)**
WP_168579423.1 SkaA *Rhodococcus* sp. DMU1	100
WP_155951766.1 SkaA *Rhodococcus aetherivorans* BCP1	99.1
WP_161572325.1 SkaA *Rhodococcus ruber* R1	98.9
APT36898.1 IifC1 *Burkholderia* sp. IDO3	35.7
ASR05096.1 GrIndA *Gordonia rubripertincta* CWB2	35.7
WP_010594217.1 SkaA *Rhodococcus ruber* SJ-1	38.2
XEZ56878.1 SkaA *Acinetobacter piscicola* p38	36.9
WP_027965539.1 HhIndA *Halomonas halocynthiae* DSM14573	34.5
CAJ94554.1 CnIndA *Cupriavidus necator* H16	35.0
ADU39062.1 VpIndA2B *Variovorax paradoxus* EPS	34.2
WP_028028710.1 GnIndA *Gemmobacter nectariphilus* 15620	32.6
EEA04565.1 BH160IndA *Burkholderia* sp. H160	33.0
EJJ03822.1 SaIndA1 *Streptomyces auratus* AGR0001	35.1
WP_039783212.1 HhMO *Herbaspirillum huttiense* subsp. *putei* IAM 15032	31.9
ACR43973.1 RoIndA1 *Rhodococcus opacus* 1CP	33.3
KWR77134.1 CSHEIndA *Cupriavidus* sp. SHE	33.3
AXK62974.1 IifC2 *Burkholderia* sp. IDO3	32.2
BAD56093.1 NfIndA1 *Nocardia farcinica* IFM 10152	33.5
ADU39063.1 VpIndA1 *Variovorax paradoxus* EPS	34.7
ABM07034.1 PaIndA1 *Paenarthrobacter aurescens* TC1	32.3
XEZ56851.1 IndA *Acinetobacter piscicola* p38	31.6
ARO76328.1 IifC *Acinetobacter* sp. O153	30.4
ENW75087.1 IifC *Acinetobacter baumannii* ATCC 19606	31.6
WP_081399512.1 IifC *Acinetobacter* sp. JW	30.5
CAG69430.1 AbIndA *Acinetobacter baylyi* ADP1	30.4
ACR43974.1 RoIndA2B *Rhodococcus opacus* 1CP	32.1
ABQ12175.1 MoxY uncultured bacterium	32.8
ABX34433.1 DaIndA *Delftia acidovorans* SPH-1	32.3
EJJ03821.1 SaIndA2B *Streptomyces auratus* AGR0001	31.6
BAD56094.1 NfIndA2B *Nocardia farcinica* IFM 10152	32.2
ABM10099.1 PaIndA2B *Paenarthrobacter aurescens* TC1	30.3
ABZ79366.1 MoxA uncultured bacterium	27.7
ABH00103.1 RjStyA *Rhodococcus jostii* RHA1	29.4
WP_027934242.1 AtStyA *Amycolatopsis thermoflava* N1165	27.7
WP_019810476.1 ShStyA *Saccharomonospora halophila* 8	29.6
ABH00101.1 RjStyA *Rhodococcus jostii* RHA1	27.4
WP_091504755.1 AsMO *Amycolatopsis sacchari* DSM 44468	28.0
WP_030740546.1 StrepStyA *Streptomyces* sp. NRRL S-31	27.3
ABV24041.1 SmoA uncultured	25.6
GAB22407.1 GpStyA *Gordonia polyisoprenivorans* NBRC 16320	25.4

BAL04132.1 RoST5StyA *Rhodococcus* sp. ST-5	29.0
BAL04129.1RoST10StyA *Rhodococcus* sp. ST-10	27.3
ASR05591.1 GrStyA *Gordonia rubripertincta* CWB2	28.7
WP_022977994.1 NrStyA *Nevskia ramosa* DSM11499	26.7
GAC06215.1 PaStyA *Paraglaciecola agarilytica* NO2	25.7
WP_039579272.1 SfStyA *Sphingopyxis fribergensis* Kp5.2	27.3
ANS32444.1 RoStyA *Rhodococcus opacus* 1CP	27.8
WP_027855270.1 MlStyA *Marinobacterium litorale* DSM 23545	24.4
CAA04000.1 PY2StyA *Pseudomonas* sp. Y2	24.0
ADE62390.1 PLQ26StyA *Pseudomonas* sp. LQ26	25.3
CAA73790.1 PpStyA S12 *Pseudomonas putida* S12	24.1
AAC23718.1 PtStyA *Pseudomonas* sp. VLB120	25.1
ABX24519.1 PpStyA CA-3 *Pseudomonas putida* CA-3	24.1
CAB06823.1 PfStyA *Pseudomonas fluorescens* ST	23.8
ABB03727.1 PpStyA SN1 *Pseudomonas putida* SN1	24.0

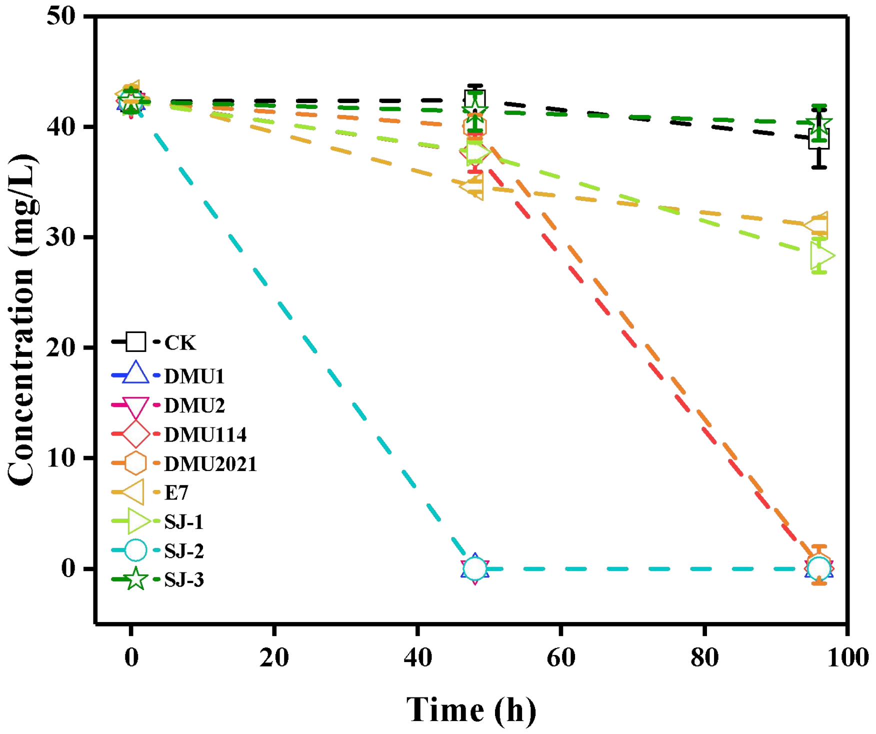

Fig. S1 Skatole-degrading curves of *Rhodococcus* strains in MS medium with skatole as the sole carbon source.

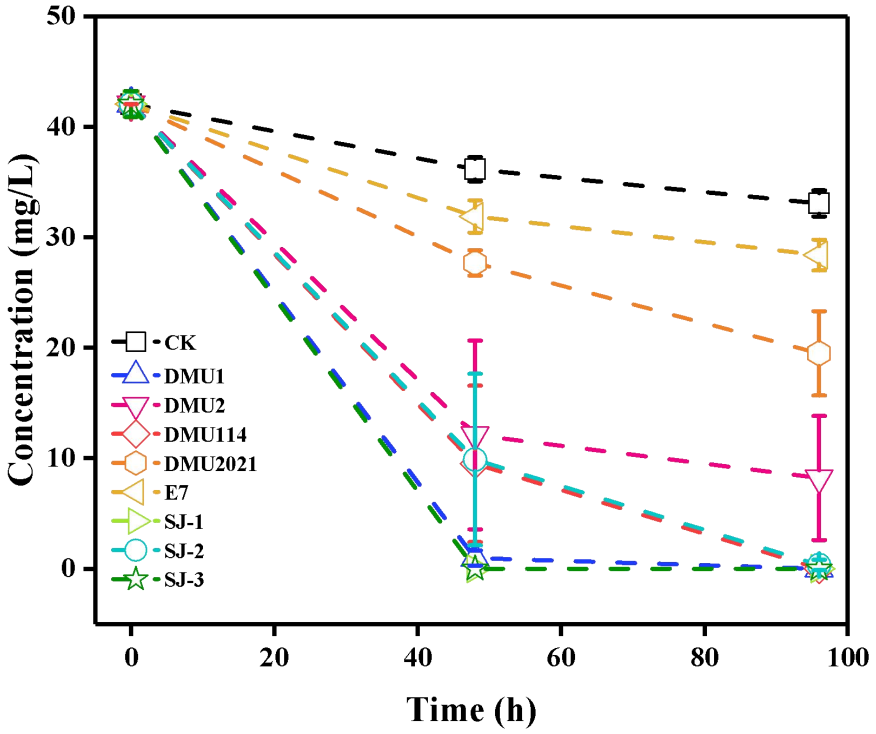

Fig. S2 Skatole-degrading curves of *Rhodococcus* strains in MSY medium with yeast extract as the extra carbon source.

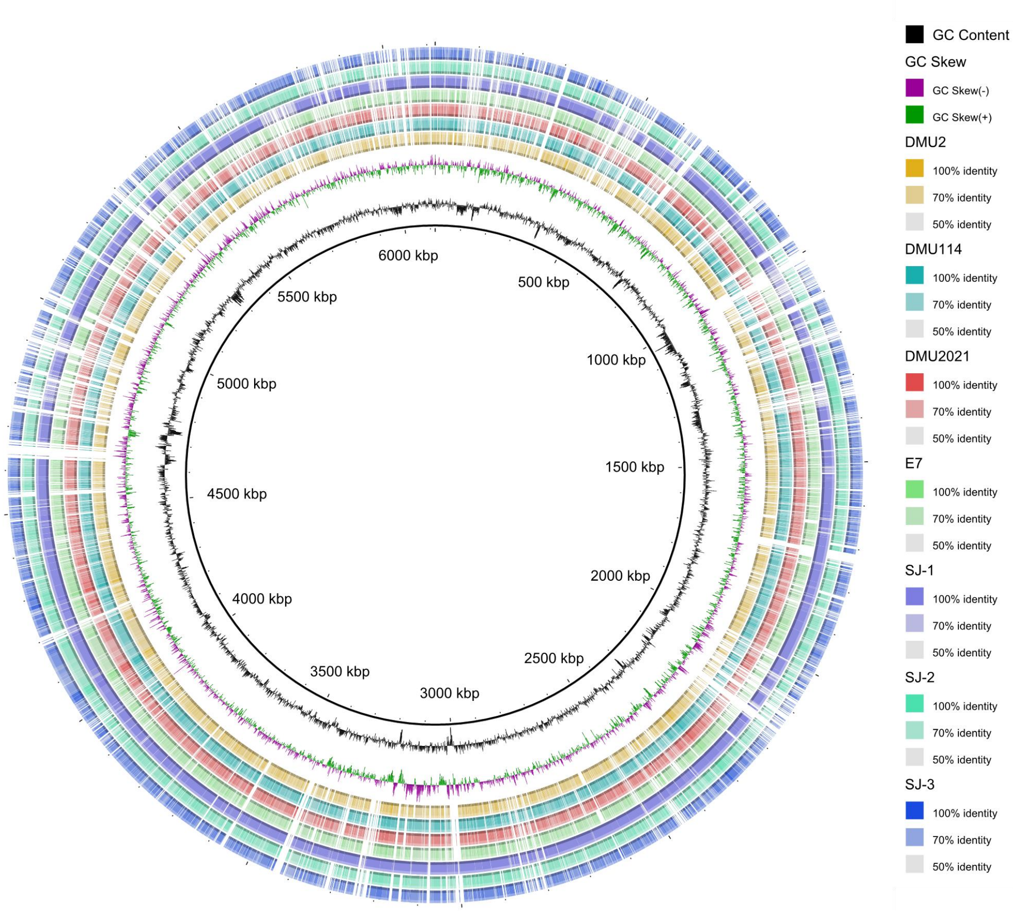

Fig. S3 Comparative genomic circular diagram of the *Rhodococcus* strains. Strain DMU1 was set as the reference.

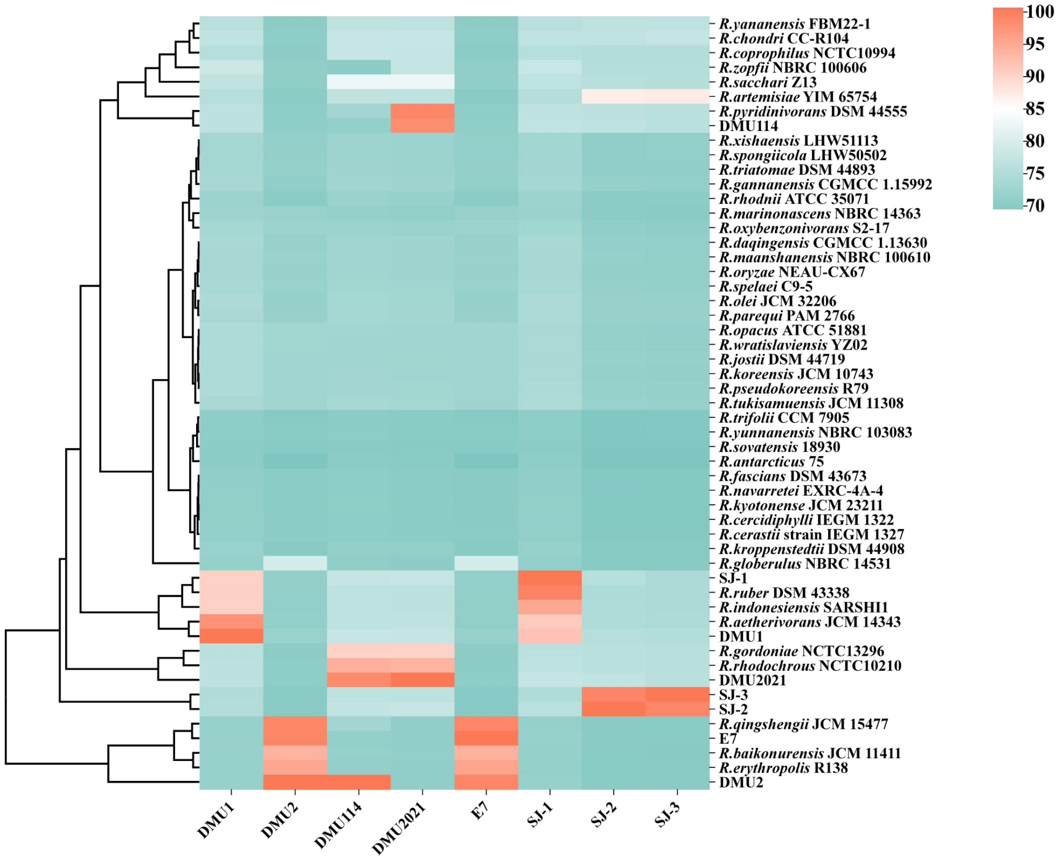

Fig. S4 Average nucleotide identity (ANI) analysis of our strains with the genomes of valid *Rhodococcus* species.

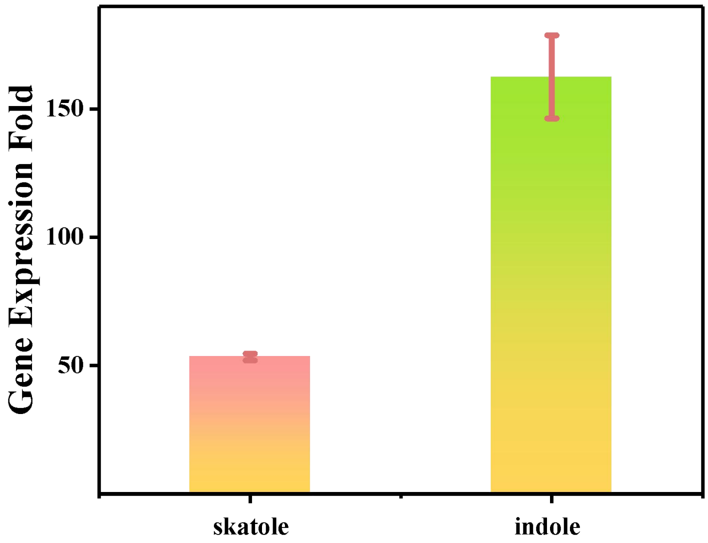

Fig. S5 qPCR results of gene *skaA* in strain DMU1 in response to skatole and indole.

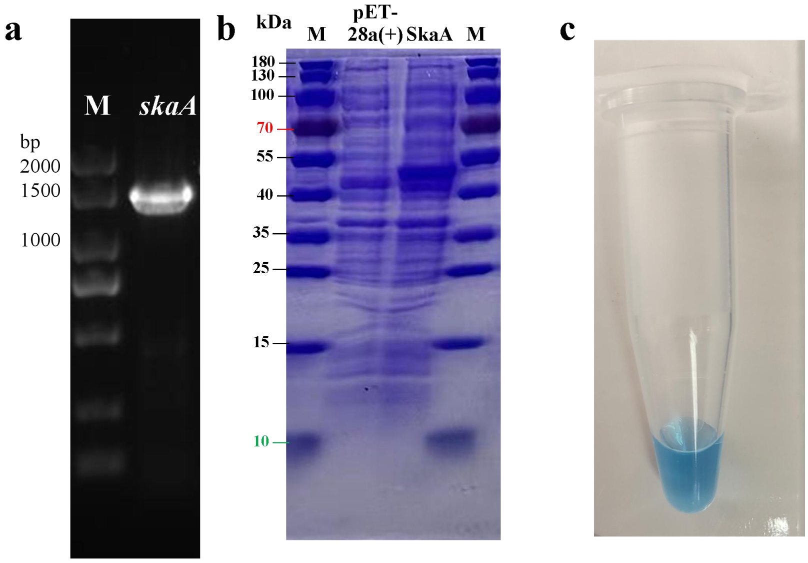

Fig. S6 Gene amplification and expression results. (a) PCR products of gene *skaA*. (b) SDS-PAGE of *E. coli* BL21(DE3) expressing *skaA* gene. (c)Indigoids formation with indole as the substrate.

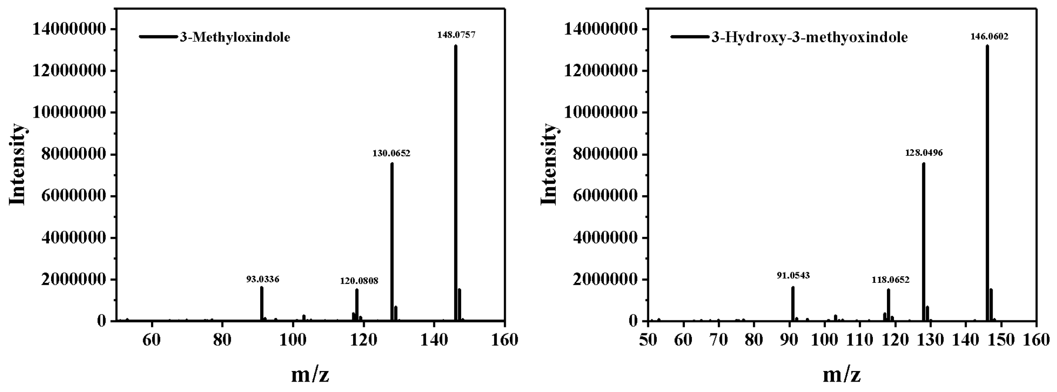

Fig. S7 Mass spectra of the standard compounds of (a) 3-methyloxindole and (b) 3-hydroxy-3-methyloxindole.

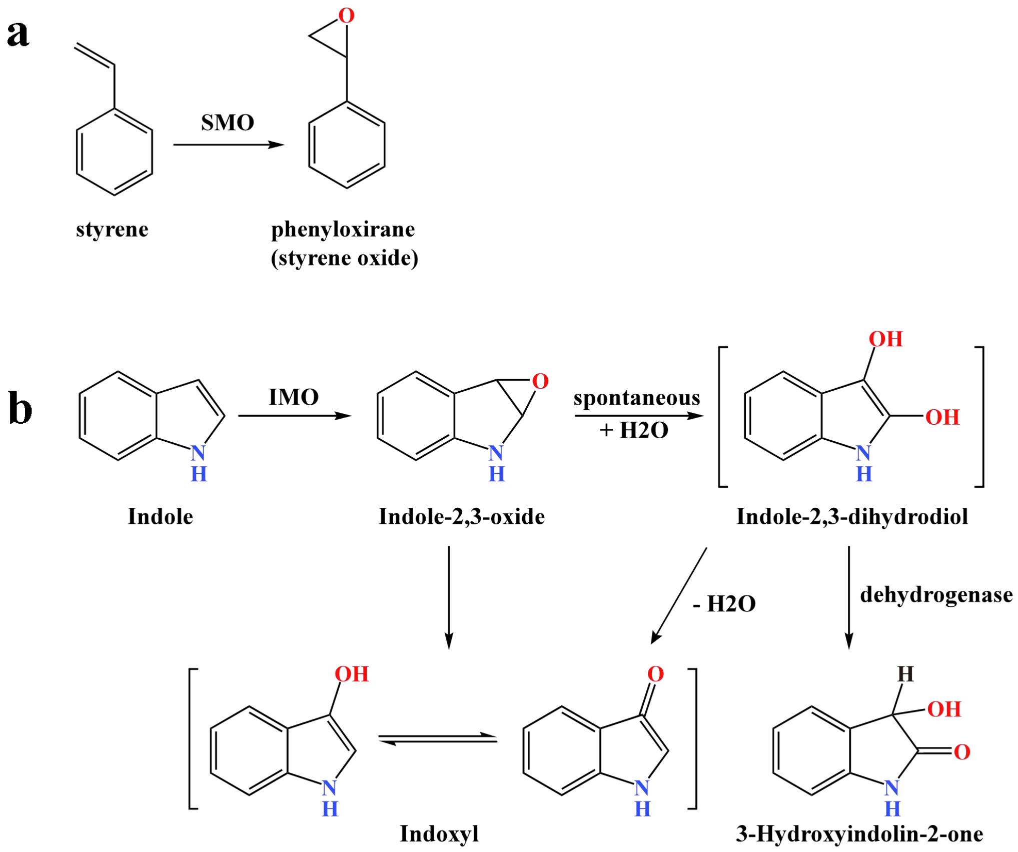

Fig. S8 Typical reactions catalyzed by (a) styrene monooxygenase (SMO) and (b) indole monooxygenase (IMO). Indole is oxidized by an IMO to form indole-2,3-epoxide, which is supposed to be spontaneously hydrolyzed to the unstable intermediate indole-2,3-dihydrodiol. This intermediate is subsequently dehydrated to yield indoxyl (Tischler et al.,The Enzymes, 2020, 47: 399-425; Heine et al., Biological Chemistry, 2019, 400(7): 939-950). Alternatively, Indole-2,3-dihydrodiol is most likely converted to 3-hydroxyindolin-2-one by a short-chain dehydrogenase (Sadauskas et al., Applied and Environmental Microbiology, 2017, 83(19): e01453-17).

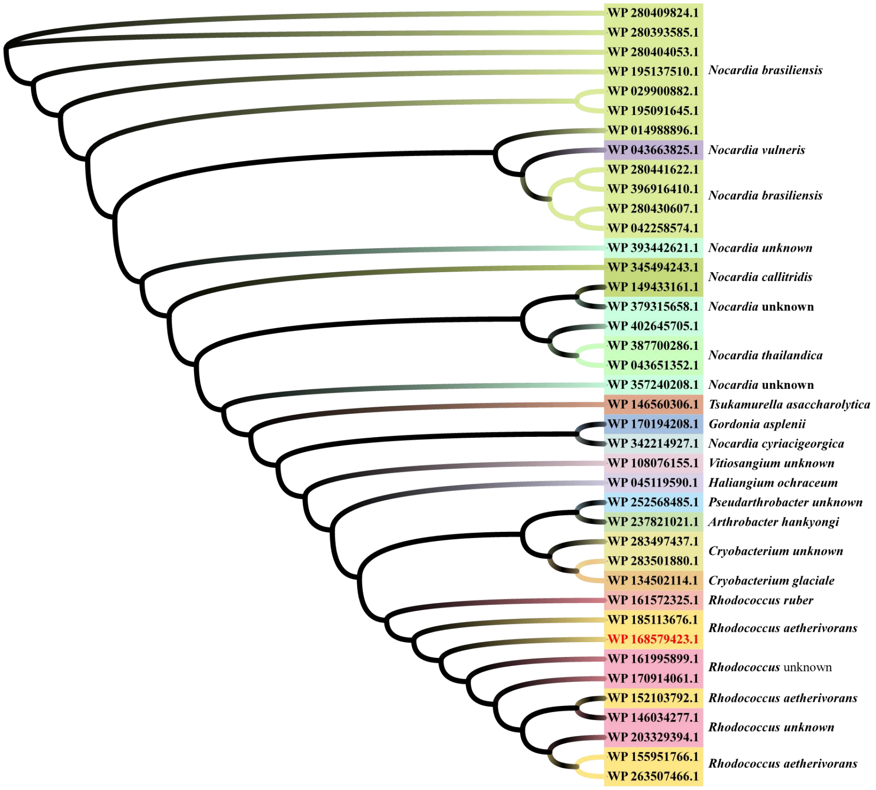

Fig. S9 Phylogenetic tree of the SkaA homologs in related strains in the RefSeq database (> 40% identity).

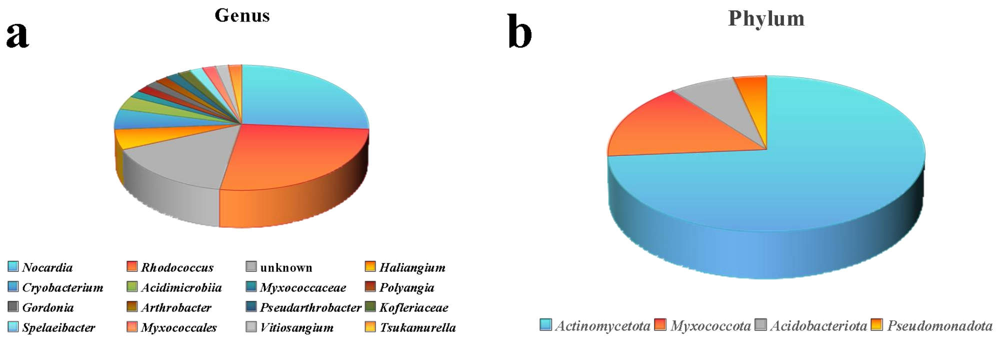

Fig. S10 Distribution of SkaA homologs in related strains in the NCBI non-redundant (NR) database. (a) Genus. (b) Phylum.

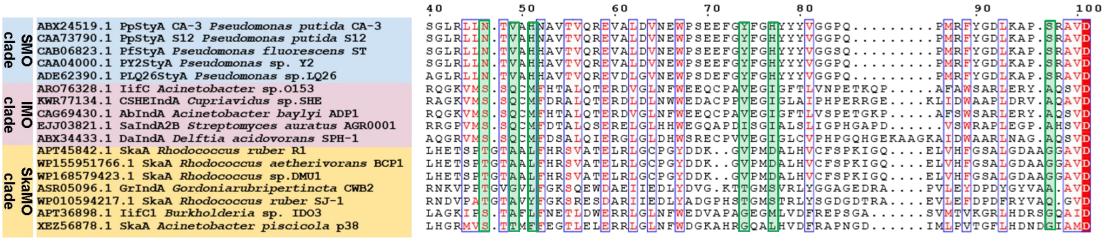

Fig. S11 Sequence alignment of typical IMOs, SMOs, and SkaMOs. The specific fingerprint residues are reported in SMO (N46-V48-H50-Y73-H76-S96) and IMO (S46-Q48-M50-V/I73-I76-A96).

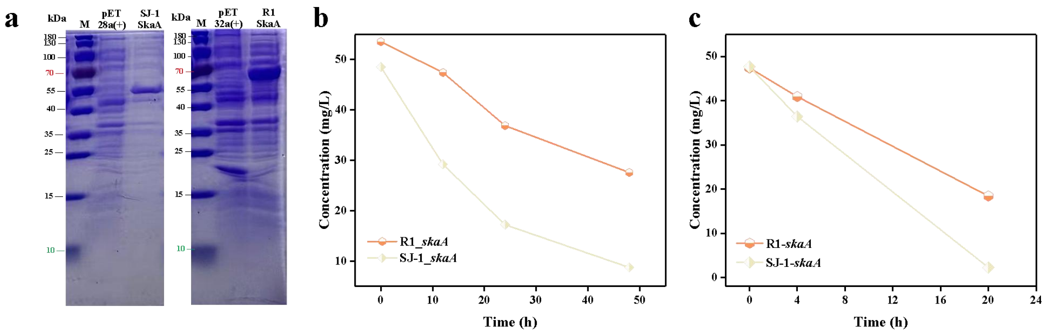

Fig. S12 Gene expression and skatole degradation performance of SkaA homologs. (a) Expression of SkaA homologs in strains SJ-1 and R1. (b) Skatole degradation in the growth culture system. (c) Skatole degradation in the resting cell system.
